# The impact of early childhood education and care‐based interventions on child physical activity, anthropometrics, fundamental movement skills, cognitive functioning, and social–emotional wellbeing: A systematic review and meta‐analysis

**DOI:** 10.1111/obr.13852

**Published:** 2024-11-07

**Authors:** Alice Grady, Rebecca Lorch, Luke Giles, Hannah Lamont, Amy Anderson, Nicole Pearson, Maria Romiti, Melanie Lum, Ashleigh Stuart, Lucy Leigh, Sze Lin Yoong

**Affiliations:** ^1^ School of Medicine and Public Health University of Newcastle Newcastle Australia; ^2^ Hunter New England Population Health, Hunter New England Local Health District Newcastle Australia; ^3^ Population Health Research Group Hunter Medical Research Institute New Lambton Heights Australia; ^4^ National Centre of Implementation Science (NCOIS) University of Newcastle Newcastle Australia; ^5^ Global Centre for Preventive Health and Nutrition, Institute for Health Transformation Deakin University Geelong Australia; ^6^ Data Sciences Hunter Medical Research Institute New Lambton Heights NSW Australia

**Keywords:** early childhood education and care, intervention, physical activity, systematic review

## Abstract

This review assessed the effectiveness of ECEC‐based interventions to improve child physical activity, and intervention impact on child weight‐based anthropometrics, fundamental movement skills (FMS), cognitive functioning, and social–emotional wellbeing. Adverse effects and costs were assessed. Finch et al's 2014 systematic review was updated. Electronic databases were searched 10 September 2014 to 27 October 2022. Included studies were randomized controlled trials of ECEC interventions targeting physical activity among children aged 0–6 years. The methodological quality of studies was assessed using Cochrane's Risk of Bias tool v2. Standardized mean differences (SMD) were calculated for each outcome with meta‐analysis undertaken; otherwise, findings were described narratively. Fifty‐three studies were included. ECEC‐based interventions were found to significantly improve child physical activity (SMD 0.193, 95% confidence interval [CI] 0.09 to 0.3; p < 0.001) and FMS (SMD 0.544, 95% CI 0.1 to 0.98; p = 0.015), compared to control. Small positive, but non‐significant, effects were found for weight‐based anthropometrics, cognitive functioning, and social–emotional wellbeing. Few studies reported adverse effects (n = 10), and no studies reported formal economic analyses. While ECEC‐based interventions can significantly improve child physical activity and FMS, further evidence of their impact on cognitive functioning, social–emotional wellbeing, and the cost‐effectiveness of such interventions is required to inform policy and practice.

**Abbreviations**
ECEC: Early childhood education and care
FMS: Fundamental movement skills
RCT: Randomized controlled trial
SMD: Standardized mean differences
MVPA: Moderate to vigorous physical activity
BMI: Body mass index
ROB2: Risk of Bias tool for randomized trials v2
CI: Confidence interval
ICC: Intracluster correlation coefficient
SD: Standard deviation
SES: Socioeconomic status

## 1 INTRODUCTION

Overweight and obesity continue to be a global public health issue, with prevalence rates having tripled since 1975.^1^ In 2019, an estimated 38.2 million (5.6%) children aged 5 years and under were overweight or obese.^1^ Obesity in childhood is a strong predictor of adult obesity,^2^ and a risk factor for non‐communicable diseases such as type 2 diabetes, cardiovascular disease, and certain cancers, which account for 41 million global deaths each year.^3^ Physical activity is a leading modifiable risk factor for childhood overweight and obesity,^4^ and is associated with numerous physical and psychological health benefits including improved motor skill and cognitive development, psychosocial health, and cardiometabolic health indicators.^5^, ^6^ The World Health Organization recommends a minimum of 180 minutes of physical activity per day for children aged 1–4 years, at least 60 minutes of daily moderate to vigorous physical activity (MVPA) in children aged 3–4 years,^7^ and a daily average of 60 minutes of MVPA for children 5 years and older.^8^ While this recommendation has been incorporated into national 24‐hour movement guidelines around the world,^9^ a recent systematic review including 23 countries reported only 55% of preschoolers obtained adequate amounts of physical activity.^10^

Center‐based early childhood education and care (ECEC) services (including nurseries, kindergartens, long day care, and preschools)^11^ are a crucial setting for the delivery of interventions to improve children's physical activity in line with global recommendations.^12^ ECEC services provide access to the majority (50–90%) of young children aged 0–5 years, for a significant proportion of their waking hours.^13^ The potential of this setting for improving the health of young children has been recognized by various governments internationally, with a systematic review of policies and guidelines in high‐income countries identifying 28 ECEC‐specific guidelines providing recommendations for physical activity.^14^ Despite the existence of such guidelines, research indicates that young children are not sufficiently active while in care.^15^, ^16^, ^17^

A number of interventions to improve child physical activity whilst in attendance at ECEC have been developed and evaluated. A recent umbrella review synthesized systematic review evidence of the effectiveness of interventions in the ECEC setting on the physical activity levels of children aged 0–6 years.^18^ The umbrella review included 10 systematic reviews, published between 2014 and 2020, and concluded that while the majority of included reviews found interventions delivered in ECEC improved child physical activity, further investigation of the effectiveness of specific intervention components was required to inform future policy and practice. The umbrella review synthesized reviews that included studies employing non‐randomized trial designs, conducted across various settings (e.g. ECEC, home, and health services), and used observational measures of child physical activity (e.g. Environment and Policy Assessment and Observation).^19^

In 2016, Finch et al published a systematic review and meta‐analysis describing the effectiveness of physical activity interventions implemented in center‐based ECEC services.^20^ Review authors concluded that while physical activity interventions delivered in the setting were effective in increasing child physical activity, effectiveness varied according to intervention characteristics, and evidence gaps remained for policy makers and practitioners. While a substantial number of eligible studies have been published since, there have been no systematic reviews or meta‐analyses aiming to synthesize the effectiveness of ECEC‐based physical activity interventions in improving objectively measured child physical activity. Additionally, none have summarized the evidence solely from randomized controlled trials (RCTs), which provide the most direct and highest level of evidence of intervention effectiveness.^21^ Despite the potential of systematic reviews to influence policy and practice, none have included a range of outcomes considered important to support decision‐making and guide future policy and practice. Outcomes such as the cost‐effectiveness and adverse consequences of interventions, and the potential co‐benefits of physical activity interventions in terms of child social–emotional wellbeing and cognition, have been identified as core outcomes in trials to prevent childhood obesity.^22^ Finally, no systematic reviews, nor meta‐analyses, have examined the evidence for ECEC‐based physical activity interventions against physical activity guideline recommendations for the sector^14^ to support existing recommendations and identify any evidence gaps. Given the increasing relevance of such research for policy and practice, an update of Finch et al's review^20^ which classifies interventions according to ECEC physical activity guidelines recommendations and includes a wider range of core outcomes for the prevention of childhood obesity (e.g. child anthropometrics, fundamental movement skills (FMS), cognitive functioning, and social–emotional wellbeing) is warranted.

## 2 AIMS

The primary aim of this review was to assess the effectiveness of ECEC‐based interventions to improve physical activity among children aged 0–6 years whilst in care. Secondary aims were to assess the impact of physical activity interventions delivered in ECEC services on child weight‐based anthropometric outcomes, child FMS, child cognitive functioning, and child social–emotional wellbeing outcomes. Economic evaluations and adverse effects of the interventions were also assessed.

## 3 METHODS

The review was conducted and reported in accordance with the Preferred Reporting Items for Systematic Reviews and Meta‐analyses 2020 guidelines,^23^ the Cochrane Handbook for Systematic Reviews of Interventions (Version 6.3),^24^ and Cochrane's Methodological Expectations of Cochrane Intervention Reviews (MECIR).^25^

### 3.1 Types of studies

Studies were required to employ an RCT study design, including cluster RCTs, stepped‐wedge RCTs, factorial RCTs, multiple baseline RCTs, and randomized crossover trials. These designs are recognized as the highest quality evidence to establish causality and quantify intervention effects.^21^

### 3.2 Types of participants

Participants primarily included children aged 0–6 years with no reported diseases or health problems, attending center‐based ECEC services. These services include public or privately operated facilities that provide care to children aged 0–6 years, outside of the home in licensed centers before commencing formal schooling (e.g. preschools, long day care services, and kindergartens).^11^ Given the range of secondary outcomes of interest to the review and the inclusion of interventions targeting all individuals involved in ECEC settings, participants could also include parents, carers, families, or guardians responsible for the care of children, and professionals responsible for the care provided to children whilst attending ECEC, such as service directors, educators, or other staff employed by the service.

### 3.3 Types of interventions

Interventions conducted in center‐based ECEC services with at least one component aimed at increasing physical activity among children were included. Intervention components could include (but not limited to) those that align with the physical activity guideline recommendations for ECEC previously identified by Jackson et al.^14^ These recommendations include: providing opportunities for children to be physically active; developing and adopting policies for physical activity and physical activity education programs; offer educator training to provide safe and developmentally appropriate physical activity; educators to promote the benefits of physical activity with children; limit the time children spend sitting (less is best); limit the use of screen time (less is best); support healthy sleeping habits; create an environment that promotes physical activity; and involve parents in the promotion of physical activity (including reduction of screen time and sedentary behaviors). See Table S1 (supporting information) for guideline‐recommended practices and example intervention components. Interventions could be delivered by (but not limited to) research staff, ECEC service staff, or any other organization or expert. Interventions that targeted both the ECEC setting and other settings, such as the home environment, were included as long as the intervention was primarily delivered in ECEC (i.e., more than 50% of the intervention components were delivered within the ECEC service, influenced the operation of the service, or both).

Interventions that targeted other health risk factors (e.g., child dietary intake) were included if the intervention contained a component that sought to influence child physical activity. Both single‐ and multi‐component interventions (i.e., interventions that included more than one component to influence child physical activity) were included.

### 3.4 Comparators/control

Studies that compared a physical activity intervention to any alternative intervention (that did not target child physical activity), usual care, ‘no intervention’ control, attention, or waitlist control were included. Studies that only compared two physical activity interventions were excluded.

### 3.5 Types of outcomes

#### 3.5.1 Primary outcome

Based on the core outcome set for early intervention trials to prevent obesity in childhood,^22^ a range of objective measures of physical activity measured via accelerometer or pedometer were included: steps or step rates, counts or count rates, minutes, time spent or percent time in physical activity (including total, light, moderate, vigorous, moderate to vigorous intensity physical activity (MVPA), leisure physical activity, or walking). Measurements taken only during attendance at ECEC or across the day (i.e., before, during, and after ECEC attendance) were included.

#### 3.5.2 Secondary outcomes

A range of secondary outcomes were included, again based on the core outcome set for early intervention trials to prevent obesity,^22^ which could be measured objectively (e.g., by trained researchers or ECEC staff) or subjectively (e.g., parent or ECEC staff reported). These included:

- Child weight status or anthropometric outcomes, including weight (kg), body mass index (BMI), BMI z‐scores, BMI percentile, weight status category (i.e., underweight, healthy weight, overweight, obese), and weight‐for‐height or weight‐for‐length;
- Child FMS, including locomotor skills (e.g., running, jumping, galloping, hopping, leaping, skipping), object control skills (e.g., throwing, catching, kicking, rolling, striking, dribbling), and stability (e.g., balance), captured by measures such as the Test of Gross Motor Development‐2^26^;
- Child cognitive functioning, including measures capturing the language and cognitive performance, such as the Bayley Scale of Infant Development^27^; Kaufman Assessment Battery for Children^28^; Wechsler Preschool and Primary Scale of Intelligence^29^; and the Early Years Toolbox^30^;
- Child social–emotional wellbeing and emotional health, including measures of social skills, problem and/or challenging behavior, self‐regulation, emotion identification and expression, social problem solving, engagement, or quality of life (e.g., Pediatric Quality of Life Inventory Generic Core Scale ‐ PedsQL™ 4.0)^31^;
- Adverse events including measures of unintended adverse effects of interventions on services, service staff, children, parents, or family. This could include impacts on child health or development (e.g., child injuries); and
- Formal economic evaluations, including any assessment of the cost‐effectiveness or cost‐utility of included interventions. Estimates of the absolute cost of an intervention (or component/s of) were excluded.

## 4 SEARCH STRATEGY

An electronic‐based literature search was performed from 10 September 2014 to 27 October 2022, to identify any relevant trials published since Finch et al's review (search period from database inception to 10 September 2014).^20^ The following electronic databases were searched: CENTRAL, CINAHL, Cochrane Reviews, Embase, Dissertations & Theses, ERIC, Medline, PsycINFO, Scopus, and SPORTDiscus.

Per the search strategy conducted by Finch et al,^20^ this updated review included filters for ‘physical activity’, ‘population’ (childcare services), and ‘interventions’. The Medline search strategy was adapted for each database using database‐specific subject headings, where available. The updated search was undertaken in consultation with an experienced medical research librarian (see supporting information S2 for electronic search strategy). Reference lists of original and newly included studies were searched for potential studies missed in the electronic database search. Reference lists of relevant systematic reviews were also searched. To identify published government reports and other gray literature we searched the World Health Organization International Clinical Trial Registry Platform,^32^ US National Institutes of Health Ongoing Trial Register, and the web engines Google and Google Scholar using the terms ‘physical activity’ and ‘childcare centre’ (of which the first 100 citations were examined). Authors of relevant protocol papers identified by the search were contacted.

Publications in peer‐reviewed journals or reports from government organizations or professional associations were included. Manuscripts or reports not published in English were excluded.

## 5 DATA EXTRACTION

### 5.1 Selection process

Pairs of reviewers (from AG, LG, RL, MR) independently screened the abstracts and titles of each article. Screening was undertaken using the online systematic review tool Covidence.^33^ Full‐text manuscripts of potentially eligible articles were obtained and independently assessed for eligibility against the inclusion criteria by pairs of reviewers (from AG, RL, HL, LG). Where differences of opinion regarding article eligibility could not be resolved through consensus, a decision was sought from a third reviewer. Reasons for study exclusion were recorded in Covidence. Authors of potentially relevant studies were not involved in screening, adjudication, data extraction, or risk of bias assessments for studies in which they were involved.

### 5.2 Data collection process

Study data was independently extracted by one reviewer (from HL, LG, RL, AA, NP) not blinded to author or journal information, and checked by a second reviewer (from AG, AA, RL, HL). A standardized and piloted data extraction form based on the Cochrane Collaboration data collection form was used. Where differences of opinion regarding data extraction could not be resolved through consensus, a decision was sought from a third reviewer (AG).

### 5.3 Data items

The following data were extracted, where available:

- Study characteristics: authors, year of publication, country, study design, and sample size;
- Setting and participant characteristics: service type, service location (urban or rural), socioeconomic characteristics (classified as low, middle, or high), and participants' ages, sex, ethnicity, and socioeconomic status (SES);
- Intervention characteristics: name of the intervention, description of intervention components, duration, delivery personnel, and setting, modality, theoretical basis for the intervention, classification according to physical activity guideline recommendations for ECEC^14^;
- Outcome measures and time points of measurement;
- Study outcomes relevant to the review, including effect size and measures of outcome variability; and
- Funding source and potential conflicts of interest.

Data items and outcomes included here that were not previously extracted by Finch et al,^20^ were also extracted from the studies originally included by Finch et al.^20^ An online graph reader^34^ was used to extract data provided only in graph format.

## 6 RISK OF BIAS ASSESSMENT

The risk of bias for each study was assessed using the revised Cochrane Risk of Bias tool for randomized trials v2 (RoB 2),^35^ described in the Cochrane Handbook for Systematic Reviews of Interventions.^36^ Assessments were undertaken on the review primary outcome only (child physical activity) across the following domains:

- Bias arising from the randomization process;
- Bias due to deviations from intended interventions;
- Bias due to missing outcome data;
- Bias in measurement of the outcome; and
- Bias in the selection of the reported result.

For cluster RCTs, the revised Cochrane risk of bias tool for cluster–randomized trials (RoB 2 CRT) was employed with an additional domain ‘bias arising from the timing of identification or recruitment of participants in a cluster randomised trial’ assessed.^35^

Pairs of reviewers (from AA, RL, NP, LG, MR, ML) independently assigned a judgment of either ‘low risk’, ‘some concerns’, or ‘high risk’ of bias for each domain and each study, according to RoB 2 algorithms and overall risk of bias judgment criteria, respectively. Where differences of opinion could not be resolved through consensus, a decision was sought from a third reviewer (AG).

## 7 DATA SYNTHESIS

The study setting, participant characteristics, outcomes, and measures of included studies were described narratively. Random effects meta‐analysis was performed on standardized mean differences (SMDs) and their variances for all primary (physical activity) and secondary (weight‐based anthropometrics, FMS, cognitive functioning, and social–emotional wellbeing) outcomes using the R package ‘metafor’ (two or more studies required per outcome).^37^ For all outcome effect sizes, we used the differences between intervention and control groups post‐intervention, in preference to the changes from baseline between the intervention and control groups, to maximize the number of studies that could be pooled.^38^ As studies used different measures or metrics to report the same outcome type, the SMD was used as the measure of effect. Statistical tests with correspondent p values or 95% confidence intervals (CI) were included where available. For cluster RCTs, reports of cluster‐adjusted analyses were used in preference to unadjusted analyses. For multi‐arm trials containing a control group and multiple interventions relevant to the review, we combined the effect estimates from multiple arms, which were then pooled with data from parallel‐arm trials.

Where multiple measures of the primary and secondary outcomes were reported in included trials, we extracted a single outcome for each study using a decision hierarchy based on outcomes that allowed us to answer the primary review aim and were most commonly reported across the included studies. We prioritized: measures with the highest quality assessment method (e.g., validated over non‐validated); measures across the day (over measures occurring only in the ECEC setting); and measures occurring immediately post‐intervention (over measures occurring during implementation).

Due to the small number of studies reporting adverse events in both intervention and control groups, substantial differences in the way adverse events were reported, and lack of formal economic evaluations, findings from these outcomes were reported narratively, using vote counting based on the direction of effect.^39^

### 7.1 Unit of analysis issues

For cluster‐RCTs that did not appropriately account for clustering, we imputed missing intracluster correlation coefficient (ICC) values with the median ICC of other included studies in this review (median ICC = 0.05^40^). For cluster‐RCTs, the trials' adjusted sample size was calculated using methods described in the Cochrane handbook before pooling with data from individual RCTs.^40^

### 7.2 Missing data

Where data essential for the calculation of an effect estimate, variability (e.g. standard deviation [SD]), or other values for inclusion in the meta‐analysis (e.g. ICC), were not reported, missing values were calculated using other reported data in the study where possible. In cases where studies did not directly report SDs, where possible these were back‐calculated using formulas outlined in the Cochrane Handbook for Systematic Reviews of Interventions from 95% Confidence Intervals (section 7.3.1), Standard Errors (section 7.5.2.3) or t‐statistics (section 7.5.2.3) if they were available.^41^ Where a study reported median and range (rather than mean and SD), the median was used as an estimate of the mean, and 1/4 × range was used as an estimate of the standard deviation.^42^ Where studies reported change from baseline within each group (rather than differences between groups at follow‐up), follow‐up means were calculated as baseline mean plus the change score and standard deviation at baseline was used to estimate standard deviation at follow‐up.^43^, ^44^ Data from intention‐to‐treat analyses were included where available.

### 7.3 Analysis of subgroups or subsets

Subgroup analyses were conducted when there were five or more studies per subgroup, per outcome type. Subgroup analyses examining the effectiveness of intervention components were undertaken. Intervention components within studies were categorized according to ECEC‐based physical activity guideline recommendations identified in Jackson et al^14^ which included:

- Provide opportunities for children to be physically active versus usual care/no intervention;
- Develop and adopt policies for physical activity and physical activity education programs versus usual care/no intervention;
- Offer educator training to provide safe and developmentally appropriate physical activity versus usual care/no intervention;
- Educators to promote the benefits of physical activity with children versus usual care/no intervention;
- Limit the time children spend sitting (less is best) versus usual care/no intervention;
- Limit use of screen time (less is best) versus usual care/no intervention;
- Support healthy sleeping habits versus usual care/no intervention;
- Create an environment that promotes physical activity versus usual care/no intervention; and
- Involve parents in the promotion of physical activity (including reduction of screen time and sedentary behaviors) versus usual care/no intervention.

Subgroup analyses examining the effectiveness of interventions primarily conducted in low SES and/or marginalized populations were also undertaken. Low SES or marginalized populations were defined as studies where the majority of participants were located in areas of low SES (as reported by study authors); studies only recruiting ECEC services participating in programs targeted at low SES populations (e.g., Head Start, Child and Adult Care Food Program) or studies targeting minority and/or marginalized groups frequently impacted by systemic inequities (e.g., Black or African Americans).

### 7.4 Assessment of heterogeneity

Potential causes of heterogeneity were evaluated via visual inspection of forest plots, with statistical heterogeneity quantified by calculating the I^2^ statistic.^45^ Where study heterogeneity was considerable (defined as I^2^ > 75%), and sufficient data was available (where there were five or more studies per subgroup), subgroup analyses according to review PICO were undertaken:

- Population: child age <2 years and child aged ≥2 years;
- Comparison: alternative intervention and usual care/waitlist;
- Outcome: outcome assessed <12 months from baseline and ≥12 months from baseline.

### 7.5 Sensitivity analysis

A sensitivity analysis to explore the impact of the study risk of bias was conducted (excluding studies classified as ‘high risk’) for the primary outcome of the review only (child physical activity).

## 8 RESULTS

### 8.1 Search results

**FIGURE 1:**
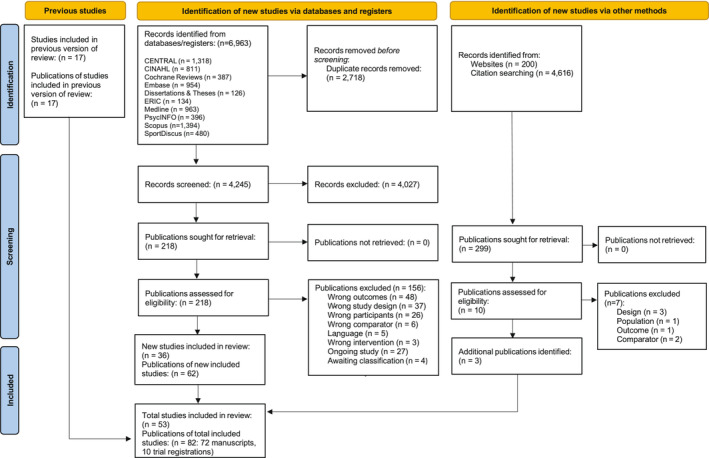
PRISMA flow diagram.

The search and selection process, including reasons for study exclusion, are illustrated in Figure 1. A total of 6963 records were identified from the electronic‐based literature search. After the removal of duplicates, the titles and abstracts of 4245 records were screened, with 218 of these assessed for eligibility. Following full‐text review, 156 records were excluded, including four studies that were unable to be classified due to limited study information^46^, ^47^, ^48^, ^49^ and 23 relevant ongoing RCTs reported across 28 protocols^50^, ^51^, ^52^, ^53^, ^54^, ^55^, ^56^, ^57^, ^58^, ^59^, ^60^ and trial registrations,^61^, ^62^, ^63^, ^64^, ^65^, ^66^, ^67^, ^68^, ^69^, ^70^, ^71^, ^72^, ^73^, ^74^, ^75^, ^76^, ^77^ which did not have published or unpublished outcomes. A total of 36 studies, reported across 62 publications were included in the electronic‐based literature search. Three further publications were identified from website searching (Google and Google Scholar), and screening of reference lists of relevant reviews and included studies. When combined with the 17 studies reported across 17 publications from Finch et al's review, a total of 53 studies reported across 82 publications were included in this review (Figure 1).

### 8.2 Included studies

Table S3 (supporting information) summarizes the characteristics of the included studies. Studies were published between 2006 and 2022. Seventeen studies were undertaken in the USA,^43^, ^44^, ^78^, ^79^, ^80^, ^81^, ^82^, ^83^, ^84^, ^85^, ^86^, ^87^, ^88^, ^89^, ^90^, ^91^, ^92^ ten in Australia,^93^, ^94^, ^95^, ^96^, ^97^, ^98^, ^99^, ^100^, ^101^, ^102^ seven in Canada,^103^, ^104^, ^105^, ^106^, ^107^, ^108^, ^109^ five in the UK^42^, ^110^, ^111^, ^112^, ^113^; two each in Belgium,^114^, ^115^ Finland,^116^, ^117^ Germany,^118^, ^119^ Greece,^120^, ^121^ and Switzerland^122^, ^123^; and one each in Ireland,^124^ Israel,^125^ and Norway.^126^ One study was conducted in multiple European countries (Belgium, Bulgaria, Germany, Greece, Poland, and Spain).^127^

One study was an RCT,^43^ with the remainder being cluster‐RCTs, one of which used a stepped‐wedge design,^98^ and two used factorial designs.^92^, ^114^ Forty‐nine studies reported one intervention group and one control group,^42^, ^43^, ^44^, ^78^, ^79^, ^80^, ^81^, ^82^, ^83^, ^84^, ^85^, ^86^, ^87^, ^88^, ^89^, ^90^, ^91^, ^93^, ^94^, ^95^, ^96^, ^97^, ^99^, ^100^, ^101^, ^102^, ^104^, ^105^, ^106^, ^107^, ^108^, ^109^, ^110^, ^111^, ^112^, ^113^, ^115^, ^116^, ^117^, ^118^, ^119^, ^120^, ^121^, ^122^, ^123^, ^124^, ^125^, ^126^, ^127^ one study included two intervention groups and one control group,^103^ and two studies included three intervention groups and one control group.^92^, ^114^ The study with a stepped‐wedge design included three intervention groups, two of which served as controls at different time points of the stepped wedge.^98^ Across all outcomes, the length of time from baseline to follow‐up ranged between two days to 15 months.

Of the 53 control groups, 37 were usual care,^42^, ^43^, ^78^, ^79^, ^80^, ^82^, ^85^, ^86^, ^87^, ^88^, ^90^, ^92^, ^95^, ^97^, ^98^, ^99^, ^100^, ^101^, ^102^, ^105^, ^107^, ^109^, ^110^, ^111^, ^112^, ^113^, ^114^, ^115^, ^116^, ^118^, ^119^, ^120^, ^121^, ^122^, ^123^, ^125^, ^127^ 14 were delayed intervention (waitlist control),^81^, ^84^, ^89^, ^91^, ^93^, ^94^, ^96^, ^103^, ^104^, ^106^, ^108^, ^117^, ^124^, ^126^ and two were alternative interventions (that did not seek to influence child physical activity).^44^, ^83^ In general, usual care included continuation of the ECEC routines as usual, which may have included the receipt of training or resources to implement health promotion programs.

#### 8.2.1 Participants

All 53 studies were conducted in center‐based ECEC services (e.g., preschool, long day care, kindergarten, nurseries). Eight studies took place in services located in urban areas,^79^, ^80^, ^84^, ^89^, ^102^, ^106^, ^110^, ^117^ five in rural areas,^42^, ^99^, ^116^, ^119^, ^123^ and eight in both urban and rural areas,^82^, ^86^, ^88^, ^96^, ^97^, ^98^, ^113^, ^114^ with the remainder of studies not reporting service location.

The number of services participating in each study ranged from 1 to 309, with the number of children ranging from 33 to 3052. Sixteen studies were considered to be large, with 400 or more children.^42^, ^44^, ^89^, ^91^, ^95^, ^99^, ^100^, ^107^, ^114^, ^115^, ^117^, ^118^, ^119^, ^122^, ^123^, ^127^ All but two studies^92^, ^125^ reported the mean age of child participants, ranging between 2.1 and 5.5 years. The proportion of female participants across 50 studies was between 36% and 61%; three studies did not report this data.^97^, ^110^, ^113^

The majority of studies did not report data to classify SES or marginalized population status. Fourteen studies^43^, ^44^, ^78^, ^82^, ^83^, ^85^, ^86^, ^88^, ^92^, ^99^, ^111^, ^112^, ^121^, ^123^ were conducted in services in low SES and/or marginalized populations, with nine of these studies including children from populations frequently impacted by systemic inequities (Aboriginal and/or Torres Strait Islander peoples, Black or African‐American, Latino or Hispanic Americans).

#### 8.2.2 Interventions

Table S1 (supporting information) summarizes the characteristics of interventions. The most common intervention characteristics included: a duration of 24 weeks/6 months (17% of studies); were conducted in the ECEC setting only (58.5%); were delivered by ECEC staff (96.2%); used face‐to‐face modalities (100%); included a component classified as ‘providing opportunities for children to be physically active’ (92.5%); and had a theoretical basis for the intervention (62.3%).

Intervention duration ranged from two days^43^ to 18 months.^99^ Interventions targeted the ECEC setting alone (31 studies),^43^, ^78^, ^79^, ^81^, ^82^, ^84^, ^85^, ^86^, ^87^, ^88^, ^89^, ^90^, ^92^, ^93^, ^94^, ^95^, ^96^, ^97^, ^100^, ^101^, ^102^, ^104^, ^105^, ^106^, ^108^, ^109^, ^112^, ^114^, ^121^, ^124^, ^126^ ECEC and home (21 studies),^42^, ^44^, ^80^, ^83^, ^91^, ^98^, ^99^, ^103^, ^107^, ^110^, ^111^, ^113^, ^115^, ^116^, ^117^, ^119^, ^120^, ^122^, ^123^, ^125^, ^127^ and one study targeted ECEC, home and community.^118^

Interventions were delivered by research staff (36 studies),^42^, ^44^, ^79^, ^80^, ^81^, ^83^, ^84^, ^85^, ^87^, ^88^, ^89^, ^91^, ^94^, ^95^, ^97^, ^98^, ^100^, ^101^, ^104^, ^107^, ^108^, ^109^, ^111^, ^113^, ^114^, ^115^, ^116^, ^117^, ^118^, ^119^, ^120^, ^121^, ^122^, ^125^, ^126^, ^127^ ECEC service staff (51 studies),^42^, ^43^, ^44^, ^78^, ^79^, ^80^, ^81^, ^82^, ^83^, ^84^, ^85^, ^86^, ^87^, ^89^, ^90^, ^91^, ^92^, ^93^, ^95^, ^96^, ^97^, ^98^, ^99^, ^100^, ^101^, ^102^, ^103^, ^104^, ^105^, ^106^, ^107^, ^108^, ^109^, ^110^, ^111^, ^112^, ^113^, ^114^, ^115^, ^116^, ^117^, ^118^, ^119^, ^120^, ^121^, ^122^, ^123^, ^124^, ^125^, ^126^, ^127^ and any other organization or expert (19 studies).^78^, ^86^, ^90^, ^99^, ^100^, ^101^, ^102^, ^103^, ^106^, ^107^, ^109^, ^110^, ^112^, ^118^, ^119^, ^120^, ^122^, ^123^, ^125^ Interventions were delivered via one or more modalities: face‐to‐face (53 studies), written (39 studies),^42^, ^44^, ^78^, ^79^, ^80^, ^81^, ^82^, ^83^, ^84^, ^86^, ^87^, ^89^, ^91^, ^93^, ^95^, ^96^, ^98^, ^99^, ^100^, ^101^, ^103^, ^104^, ^106^, ^107^, ^108^, ^110^, ^111^, ^113^, ^115^, ^116^, ^117^, ^118^, ^119^, ^121^, ^122^, ^123^, ^124^, ^126^, ^127^ online (12 studies),^80^, ^86^, ^87^, ^91^, ^98^, ^99^, ^107^, ^110^, ^113^, ^117^, ^118^, ^126^ telephone (six studies),^87^, ^95^, ^99^, ^100^, ^107^, ^110^ video (five studies),^86^, ^90^, ^96^, ^113^, ^118^ digital versatile disc (DVD)/compact disc (CD) (three studies),^44^, ^95^, ^123^ and short message service (SMS) (two studies).^87^, ^110^

In line with the physical activity guideline recommendations for ECEC,^14^ studies included intervention components that:

- Provide opportunities for children to be physically active (49 studies)^42^, ^43^, ^44^, ^78^, ^80^, ^81^, ^82^, ^83^, ^84^, ^85^, ^86^, ^87^, ^88^, ^89^, ^90^, ^91^, ^92^, ^93^, ^94^, ^95^, ^96^, ^97^, ^98^, ^99^, ^100^, ^101^, ^102^, ^103^, ^104^, ^105^, ^106^, ^107^, ^109^, ^110^, ^111^, ^112^, ^113^, ^115^, ^116^, ^117^, ^118^, ^119^, ^120^, ^122^, ^123^, ^124^, ^125^, ^126^, ^127^;
- Develop and adopt policies for physical activity and physical education programs (six studies)^95^, ^98^, ^102^, ^108^, ^110^, ^123^;
- Offer educator training to provide safe and developmentally appropriate physical activity (41 studies)^42^, ^44^, ^78^, ^79^, ^80^, ^81^, ^82^, ^83^, ^84^, ^86^, ^87^, ^89^, ^90^, ^91^, ^93^, ^95^, ^96^, ^97^, ^98^, ^99^, ^101^, ^103^, ^104^, ^106^, ^107^, ^108^, ^109^, ^110^, ^111^, ^112^, ^115^, ^116^, ^117^, ^119^, ^120^, ^121^, ^122^, ^123^, ^124^, ^126^, ^127^;
- Recommend educators promote the benefits of physical activity with children (35 studies)^79^, ^80^, ^81^, ^82^, ^84^, ^86^, ^87^, ^89^, ^90^, ^91^, ^92^, ^93^, ^94^, ^95^, ^96^, ^97^, ^98^, ^99^, ^101^, ^103^, ^104^, ^106^, ^110^, ^111^, ^112^, ^113^, ^117^, ^118^, ^119^, ^121^, ^123^, ^124^, ^125^, ^126^, ^127^;
- Limit the time children spend sitting (10 studies)^87^, ^93^, ^95^, ^98^, ^99^, ^106^, ^110^, ^111^, ^125^, ^126^;
- Limit use of screen time (10 studies)^42^, ^44^, ^95^, ^110^, ^111^, ^116^, ^117^, ^119^, ^123^, ^125^;
- Support healthy sleeping habits (three studies)^80^, ^110^, ^123^;
- Create an environment that promotes physical activity (31 studies)^42^, ^43^, ^78^, ^79^, ^83^, ^84^, ^87^, ^89^, ^90^, ^92^, ^93^, ^95^, ^96^, ^97^, ^98^, ^103^, ^104^, ^106^, ^107^, ^109^, ^110^, ^111^, ^114^, ^115^, ^116^, ^118^, ^120^, ^122^, ^123^, ^126^, ^127^; and
- Involve parents in the promotion of physical activity (18 studies).^42^, ^44^, ^80^, ^83^, ^91^, ^98^, ^99^, ^103^, ^111^, ^113^, ^116^, ^117^, ^118^, ^119^, ^120^, ^122^, ^123^, ^127^

Thirty‐three studies reported the theoretical basis of the interventions, including Socioecological Model (12 studies)^80^, ^81^, ^84^, ^89^, ^95^, ^99^, ^103^, ^107^, ^112^, ^116^, ^122^, ^123^; Social Cognitive Theory (nine studies)^44^, ^82^, ^86^, ^87^, ^93^, ^96^, ^110^, ^111^, ^117^; PRECEDE‐PROCEED Model (three studies)^109^, ^115^, ^127^; Skinnerian and social learning principles^113^; Vygotsky's Social Development Theory^101^; Wenger's Theory on Communities of Practice^126^; McLeroy's Ecological Model^104^; General Systems Theory^118^; Achievement Goal Theory^88^; Guskey's Model of Teacher Change^98^; Holistic Pedagogic Approach^119^; and Social Marketing Approach.^91^

#### 8.2.3 Outcomes

For the primary outcome, physical activity was measured using only accelerometers in 47 studies, five used only pedometers,^83^, ^95^, ^120^, ^121^, ^125^ and one used both.^127^ Studies assessed a range of continuous physical activity outcomes, including time spent in MVPA (e.g., proportion and minutes per hour/day/during care), number of steps (e.g., total in care, weekday +/− weekend), non‐sedentary physical activity, and total physical activity.

For secondary outcomes, 17 studies reported on anthropometric outcomes.^42^, ^44^, ^83^, ^91^, ^93^, ^97^, ^101^, ^103^, ^106^, ^110^, ^111^, ^113^, ^118^, ^119^, ^122^, ^123^, ^125^ All were continuous outcomes and included BMI, BMI z‐score, and BMI percentile. Two studies did not specify the measures used; the remainder used digital/electronic scales (weight), with or without stadiometers (height).

Child FMS outcomes were reported in 16 studies.^42^, ^78^, ^83^, ^88^, ^94^, ^96^, ^97^, ^103^, ^104^, ^106^, ^107^, ^119^, ^120^, ^122^, ^123^, ^124^ All were continuous outcomes and included global motor score, gross motor quotient and locomotor score, with measures including the Test of Gross Motor Development 2nd edition (TGMD‐2),^78^, ^88^, ^94^, ^96^, ^97^, ^103^, ^104^, ^107^, ^124^, ^128^, ^129^ the Peabody Developmental Motor Scales (PDMS‐2)^83^ and Korper‐Koordination Test for Kinder (KTK).^120^

Four studies reported child cognitive functioning measures,^93^, ^102^, ^117^, ^123^ with continuous outcomes of inhibition, spatial working memory, and cognitive self‐regulation (SR) skills. Measures used included the Early Years Toolbox (‘Go/No‐Go’ game),^93^, ^102^ Intelligence and Development Scales (IDS),^123^ and Cognitive SR skills sub‐dimension of the Child Social Behavior Questionnaire.^117^

Child social–emotional wellbeing and emotional health outcomes were reported in six studies.^103^, ^110^, ^117^, ^118^, ^122^, ^123^ All outcomes were continuous and included quality of life, health‐related quality of life, and emotional self‐regulation skills. The Pediatric Quality of Life Inventory (PedsQL) 4.0 measure was used by four of the six studies.^103^, ^110^, ^122^, ^123^

Adverse outcomes were reported by 10 studies in total, four of which^95^, ^100^, ^110^, ^119^ explicitly measured and reported outcomes including increased occurrence of injury among children, number of adverse events, and number of accidents via parental questionnaire and adverse event/incident report forms. An additional six studies did not report the specific measures used to assess this outcome.^80^, ^96^, ^98^, ^99^, ^123^, ^125^ One study^93^ reported to measure adverse events and injuries, but did not report findings.

Costs related to the physical activity intervention, or its implementation were available for eight studies,^42^, ^44^, ^86^, ^95^, ^107^, ^110^, ^114^, ^122^ and included total costs and cost of equipment. None of the studies undertook a formal economic evaluation (e.g., cost‐effectiveness, cost‐utility) of the physical activity intervention, and as such, findings have not been included here.

### 8.3 Risk of bias assessment

**FIGURE 2:**
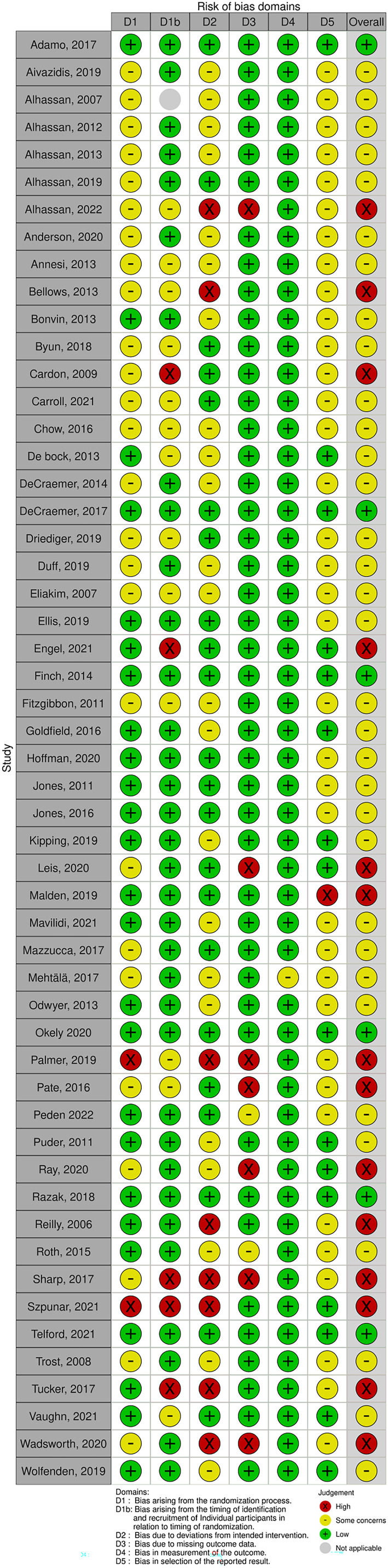
Risk of bias assessment.

For the primary outcome (child physical activity), risk of bias assessments are described in Figure 2.^130^ Overall, six studies (11.3%) were judged ‘low risk’, 33 studies (62.3%) as ‘some concerns’, and 14 studies (26.4%) as ‘high risk’.

Twenty‐six studies (49.1%) were considered to have ‘some concerns’ for bias arising from the randomization process; 34 out of 52 cluster RCTs (65.4%) were considered ‘low risk’ for bias arising from the timing of identification or recruitment of participants; 23 studies (43.4%) were considered to have ‘some concerns’ for bias due to deviations from intended interventions; 44 studies (83.0%) were considered ‘low risk’ for bias due to missing outcome data; 52 studies (98.1%) were ‘low risk’ for bias in measurement of the outcome; and 36 studies (67.9%) were considered to have ‘some concerns’ for bias in selection of the reported result.

### 8.4 Effectiveness of interventions

#### 8.4.1 Primary outcome ‐ child physical activity

**FIGURE 3:**
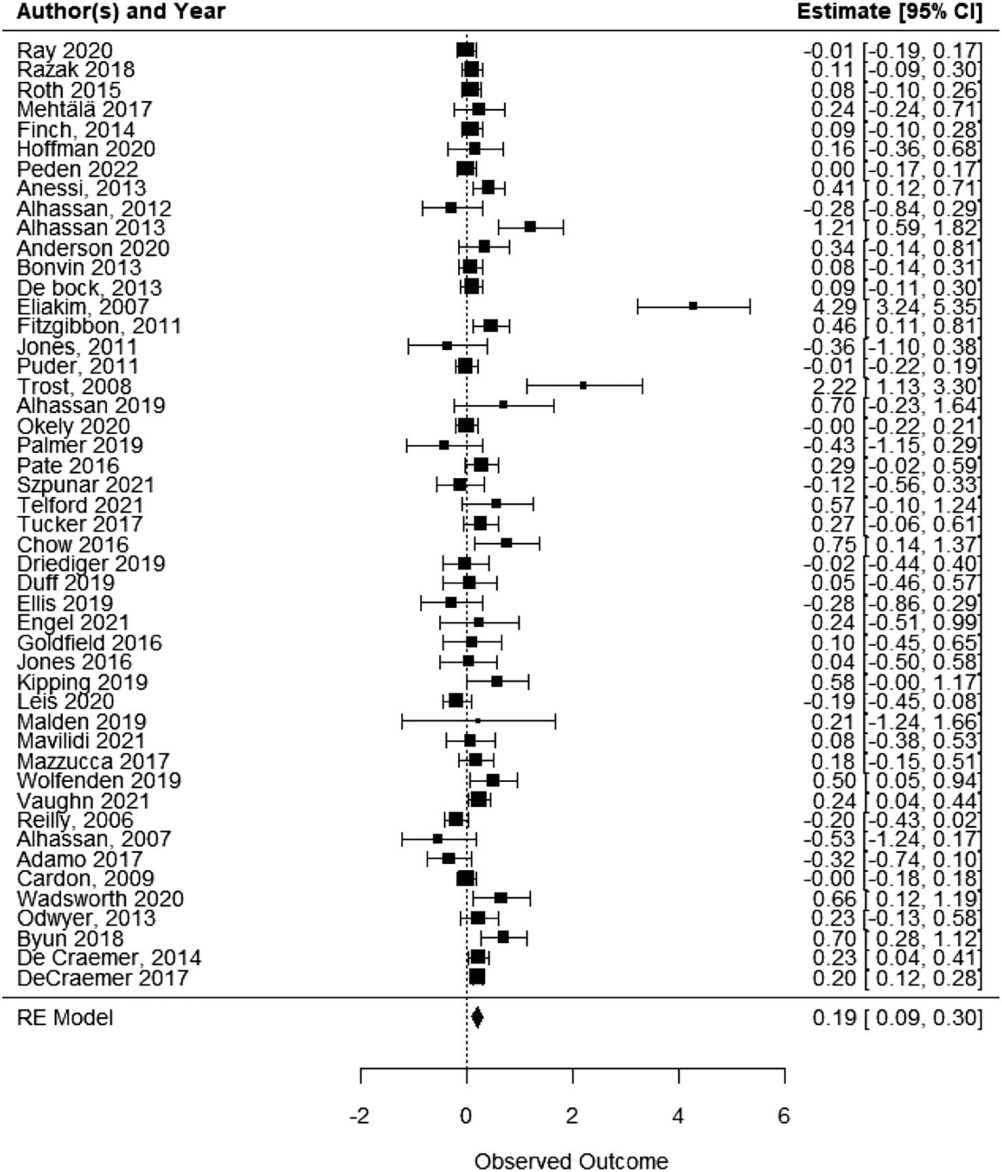
Child physical activity forest plot.

All included studies (n = 53) reported on some measure of physical activity when comparing an ECEC‐based physical activity intervention with a usual care/waitlist/alternative intervention. Five studies were excluded from the meta‐analysis due to insufficient data: two reported change scores and their SD, with no baseline mean or SD,^81^, ^113^ two had no measure of variance,^85^, ^120^ and the remaining one did not report any effect size.^83^ Pooled analysis of 48 studies indicated that ECEC‐based physical activity interventions increased child physical activity relative to control (SMD 0.193, 95% CI 0.09 to 0.3; p < 0.001, I^2^ = 82.2%; 13,283 children) (Figure 3). A SMD of 0.193 is equivalent to an increase of 3.54 (95% CI 1.58 to 5.51) minutes of MVPA per day. In the sensitivity analysis which excluded 11 studies judged as overall high risk of bias, results remained statistically significant (SMD 0.263, 95% CI 0.11 to 0.42; p = 0.001, I^2^ = 89.96%; 10,641 children).

#### 8.4.2 Subgroup analyses

In the subgroup analyses by intervention classification, a significant positive effect on child physical activity was found for interventions that included: providing opportunities for children to be physically active (SMD 0.187, 95% CI 0.08 to 0.29; p < 0.001, I^2^ = 79.73%; 44 studies; 12,355 children), offering educator training (SMD 0.152, 95% CI 0.07 to 0.23; p < 0.001, I^2^ = 60.34%; 38 studies; 11,155 children), educators promoting the benefits of physical activity (SMD 0.329, 95% CI 0.12 to 0.53; p = 0.002, I^2^ = 93.18%; 32 studies; 9520 children), creating an environment that promotes physical activity (SMD 0.147, 95% CI 0.03 to 0.26; p = 0.01, I^2^ = 74.75%; 29 studies; 8606 children), and parental involvement (SMD 0.082, 95% CI 0 to 0.16; p = 0.048, I^2^ = 47.07%; 16 studies; 7706 children) (Table 1).

**TABLE 1:** Results of subgroup meta‐analyses by intervention classification.

Intervention	SMD	95% CI	p‐value	I‐squared	Tau‐squared	Number of studies	Number of children
**Child physical activity**							
Develop and adopt policies for physical activity	0.058	‐0.04 to 0.16	0.263	0.00	0.00	6	1985
Provide opportunities for physical activity	0.187	0.08 to 0.29	0.000528	79.73	0.08	44	12,355
Offer educator training	0.152	0.07 to 0.23	0.000262	60.34	0.03	38	11,155
Educators promote the benefits of physical activity	0.329	0.12 to 0.53	0.0016	93.18	0.28	32	9520
Limit the time children spend sitting	0.501	−0.25 to 1.25	0.19	97.97	1.35	10	2104
Limit the use of screen time	0.517	−0.22 to 1.25	0.169	98.59	1.32	10	2905
Environment that promotes physical activity	0.147	0.03 to 0.26	0.0118	74.75	0.06	29	8606
Involve parents in the promotion of physical activity	0.082	0 to 0.16	0.0484	47.07	0.01	16	7706
**Child weight status/anthropometric outcomes**							
Provide opportunities for physical activity	−0.015	−0.08 to 0.05	0.664	0.03	0.00	16	5351
Offer educator training	0.016	−0.06 to 0.09	0.655	0.00	0.00	13	4554
Educators promote the benefits of physical activity	−0.055	−0.29 to 0.18	0.645	85.30	0.13	13	3714
Limit the time children spend sitting	−0.316	−1.13 to 0.5	0.449	89.28	0.77	5	426
Limit the use of screen time	−0.226	−0.73 to 0.28	0.383	95.54	0.42	7	2537
Environment that promotes physical activity	−0.016	−0.11 to 0.08	0.741	0.00	0.00	10	2806
Involve parents in the promotion of physical activity	−0.008	−0.08 to 0.06	0.815	0.00	0.00	11	4691
**Child fundamental movement skills**							
Provide opportunities for physical activity	0.544	0.1 to 0.98	0.0152	95.80	0.72	16	3899
Offer educator training	0.369	−0.01 to 0.75	0.0541	94.00	0.44	14	3762
Educators promote the benefits of physical activity	0.116	0.01 to 0.22	0.034	0.01	0.00	9	1903
Environment that promotes physical activity	0.405	−0.05 to 0.86	0.0832	94.28	0.57	12	3067
Involve parents in the promotion of physical activity	0.538	−0.26 to 1.34	0.188	98.26	1.13	7	2720
**Child social–emotional wellbeing**							
Provide opportunities for physical activity	0.059	−0.03 to 0.15	0.215	0	0	5	2461
Involve parents in the promotion of physical activity	0.059	−0.03 to 0.15	0.215	0	0	5	2461

The subgroup analysis with interventions undertaken in low SES and/or marginalized populations did not have a significant effect on physical activity (SMD 0.123, 95% CI − 0.04 to 0.29; p = 0.15, I^2^ = 50.44%; 12 studies; 2267 children). However, when analyses were limited to interventions conducted in high, mixed, or unclear SES populations (i.e., not in low SES and/or marginalized populations) the significant effect on physical activity remained (SMD 0.253, 95% CI 0.09 to 0.41; p = 0.002, I^2^ = 91.24%; 36 studies; 11,016 children).

As heterogeneity was high (I^2^ > 75%) further subgroup analyses were undertaken. The subgroup analyses with outcomes measured <12 months post‐baseline (SMD 0.209, 95% CI 0.06 to 0.35; p = 0.005, I^2^ = 86.11%; 42 studies; 9291) and ≥12 months post‐baseline (SMD 0.184, 95% CI 0.12 to 0.25; p < 0.001, I^2^ = 0.0%; 6 studies; 3992) remained significant. Subgroup analyses by population and comparison were not performed due to the insufficient number of studies.

### 8.5 Secondary outcomes

#### 8.5.1 Child weight status or anthropometric outcomes

**FIGURE 4:**
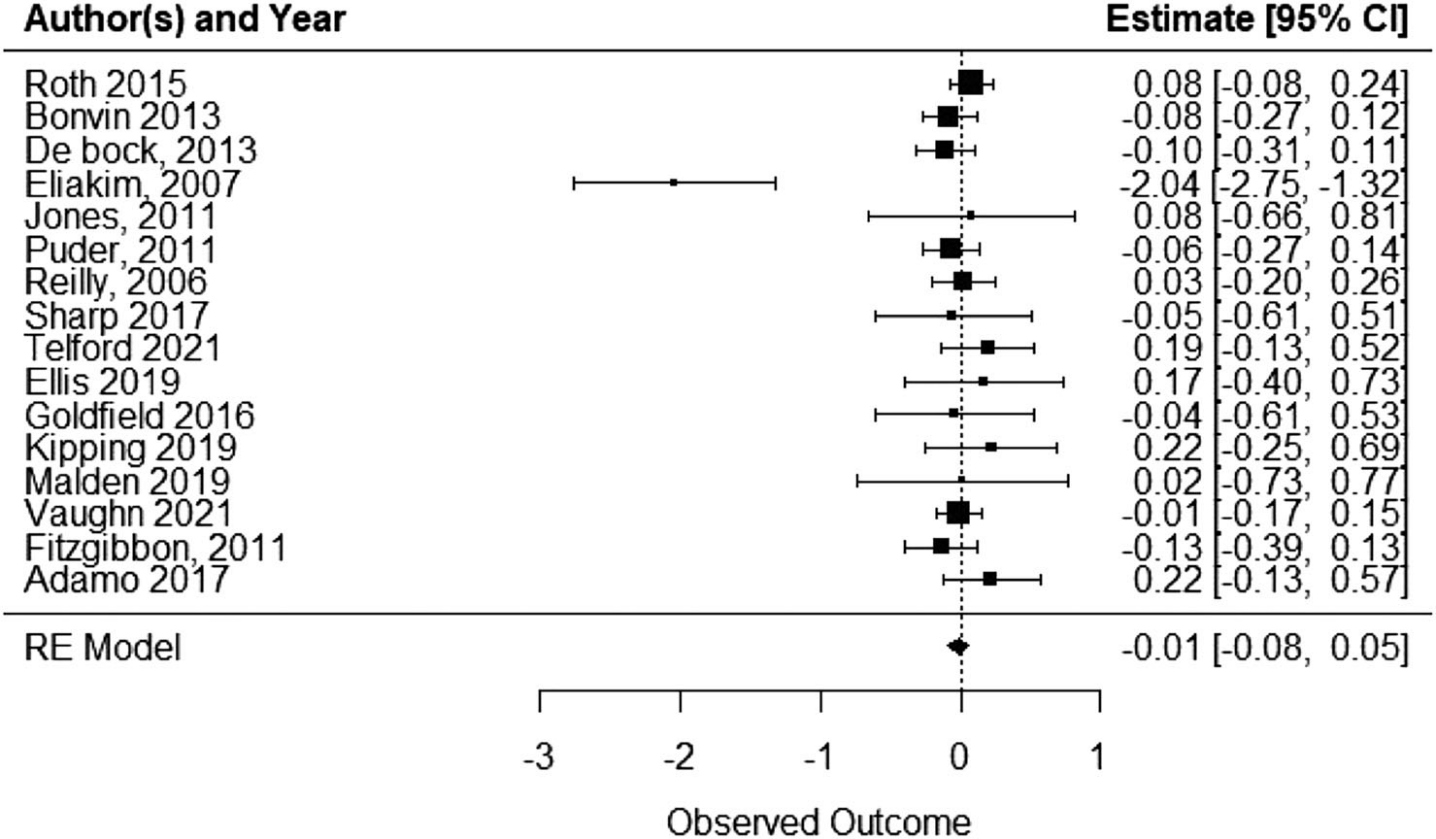
Child weight status or anthropometric outcomes forest plot.

A total of 17 included studies reported on some measure of child weight status or anthropometric outcome when comparing an ECEC‐based physical activity intervention with a usual care/waitlist/control. One study^83^ did not report any effect size, so was excluded from the meta‐analysis. Pooled analysis of 16 studies indicated that ECEC‐based physical activity interventions slightly reduced child weight status/anthropometric outcomes relative to control, however, this was not statistically significant (SMD ‐0.015, 95% CI ‐0.08 to 0.05; p = 0.664, I^2^ = 0.03%; 16 studies; 5351 children) (Figure 4). A SMD of −0.015 is equivalent to a reduction of 0.02 (95% CI –0.13 to 0.08) in BMI score.

#### 8.5.2 Subgroup analyses

In the subgroup analyses by intervention classification, small but non‐significant reductions in child weight status/anthropometric outcomes were found for providing opportunities for children to be physically active, educators promoting the benefits of physical activity, limiting sitting time, limiting screen time, creating an environment that promotes physical activity, and parental involvement (Table 1).

Only two studies investigated the effects of supporting healthy sleep habits and developing and adopting standards for physical activity on child measures of child weight status/anthropometrics, and only four studies investigated the effects of physical activity interventions on anthropometric outcomes in low SES and/or marginalized populations, therefore meta‐analyses were not performed on these groups.

### 8.6 Child fundamental movement skills

**FIGURE 5:**
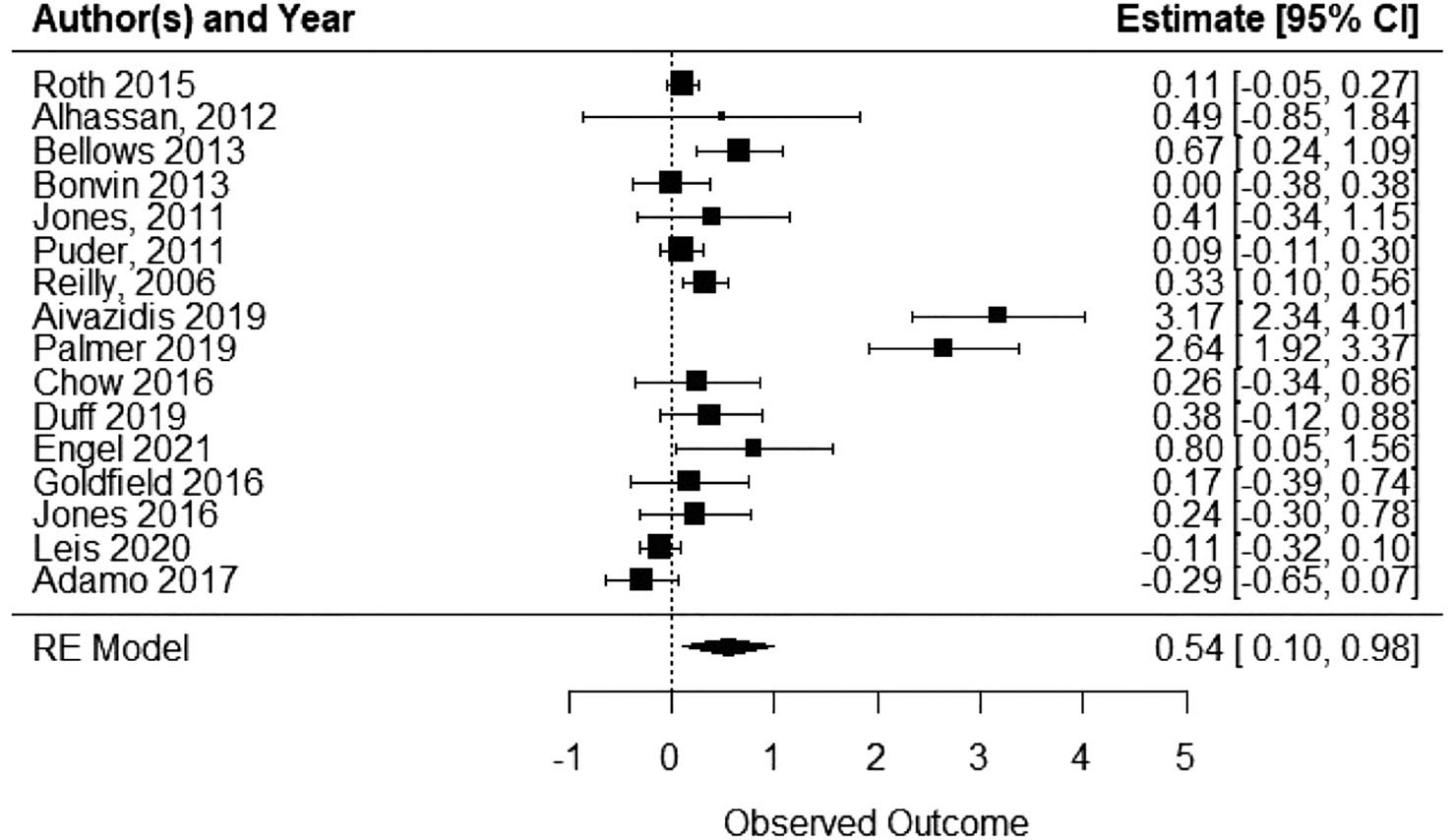
Child fundamental movement skills forest plot.

A total of 16 included studies reported on some measure of child FMS when comparing an ECEC‐based physical activity intervention with a usual care/waitlist/control. Pooled analysis of 16 studies indicated that ECEC‐based physical activity interventions increased child FMS outcomes relative to control (SMD 0.544, 95% CI 0.1 to 0.98; p = 0.015, I^2^ = 95.8%; 16 studies; 3899 children) (Figure 5). A SMD of 0.544 is equivalent to an increase of 1.55 (0.3 to 2.8) global motor score points.

#### 8.6.1 Subgroup analyses

In the subgroup analyses by intervention classification, interventions that involved providing opportunities for children to be physically active (SMD 0.544, 95% CI 0.1 to 0.98; p = 0.0152, I^2^ = 95.80%; 16 studies; 3899 children) and educators promoting the benefits of physical activity (SMD 0.116, 95% CI 0.01 to 0.22; p = 0.034, I^2^ = 0.01; 9 studies; 1903 children), had a statistically significant effect on FMS (Table 1). Non‐significant positive effects were found for interventions including educator training, creating an environment that promotes physical activity, and parental involvement (Table 1).

As less than five studies included developing and adopting policies for physical activity (n = 0), supporting healthy sleep habits (n = 1), limiting screen time (n = 2), limiting sitting time (n = 1), and investigating the effects of interventions on child FMS outcomes in low SES and/or marginalized populations (n = 4), meta‐analyses for these subgroups were not performed.

Due to an insufficient number of studies, further subgroup analyses investigating heterogeneity were not performed.

### 8.7 Child cognitive functioning

**FIGURE 6:**
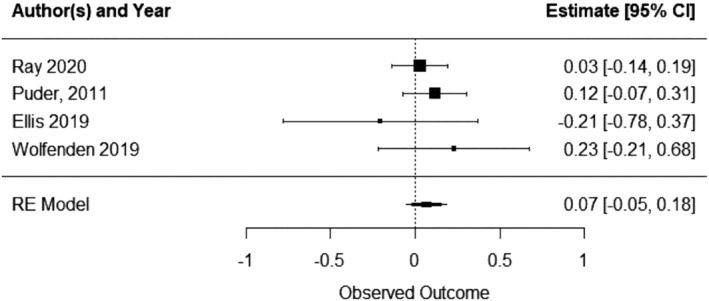
Child cognitive functioning forest plot.

A total of four included studies reported on some measure of child cognitive functioning when comparing an ECEC‐based physical activity intervention with a usual care/waitlist/control. Pooled analysis of four studies indicated that ECEC‐based physical activity interventions did not produce statistically significant increases in child cognitive functioning relative to control (SMD 0.066, 95% CI ‐0.05 to 0.18; P = 0.268, I^2^ = 0; 4 studies; 1525 children) (Figure 6). A SMD of 0.066 is equivalent to an increase of 0.03 (95% CI –0.02 to 0.08) cognitive self‐regulation skills points.

Due to the small number of studies included in the meta‐analysis, subgroup analyses were not undertaken.

### 8.8 Child social–emotional wellbeing

**FIGURE 7:**
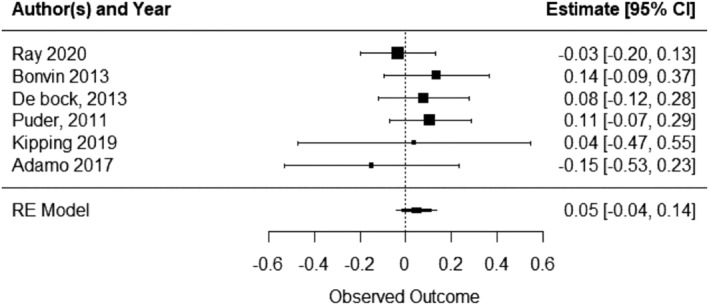
Child social–emotional wellbeing forest plot.

A total of six included studies reported on some measure of child social–emotional wellbeing when comparing an ECEC‐based physical activity intervention with a usual care/waitlist/control. Pooled analysis indicated that ECEC‐based physical activity interventions result in small positive, but not statistically significant increases in child social–emotional wellbeing relative to control (SMD 0.047, 95% CI ‐0.04 to 0.14; P = 0.304, I^2^ = 0; 6 studies; 2676 children) (Figure 7). A SMD of 0.047 is equivalent to an increase of 0.02 (95% CI –0.02 to 0.05) quality of life points.

#### 8.8.1 Subgroup analyses

In the subgroup analyses by intervention classification, five studies that investigated child social–emotional wellbeing included intervention components classified as providing opportunities for children to be physically active and parental interventions to increase physical activity. A small positive, but not statistically significant effect was found for these studies compared to control (Table 1). As less than five studies included the remaining intervention components, further subgroup analyses were not performed.

Only one study investigated the effects of physical activity interventions on child social–emotional wellbeing outcomes in low SES and/or marginalized populations, therefore meta‐analysis was not performed.

### 8.9 Adverse events

Ten studies reported on the adverse effects of the physical activity intervention.^80^, ^95^, ^96^, ^98^, ^99^, ^100^, ^110^, ^119^, ^123^, ^125^ Seven studies stated that no adverse events, consequences, or injuries were reported as a result of the intervention,^80^, ^96^, ^98^, ^99^, ^110^, ^123^, ^125^ however did not provide any numerical data to compare findings between intervention and control groups. The remaining three studies^95^, ^100^, ^119^ were deemed inappropriate for pooling due to substantial differences in the way the outcomes were reported. Finch et al, reported on participating ECEC's injury rate (per month), Razak et al reported on the number of injuries across the study period per ECEC (3 months), and Roth et al reported the frequency of accidents for participating children (time frame not provided). All three studies reported no significant differences between groups, two of which showed no negative effects in the intervention group compared to control.^95^, ^119^

### 8.10 Formal economic evaluations

None of the included studies undertook formal economic evaluations of the physical activity intervention.

## 9 DISCUSSION

This review of 53 RCTs suggests physical activity interventions delivered in ECEC settings can significantly improve child physical activity. The effects of the interventions on child physical activity increased slightly in the sensitivity analysis (from SMD 0.193 to 0.263), which excluded 11 studies with a high risk of bias. The effects reported here are consistent with, albeit smaller than, an earlier version of this review by Finch and colleagues,^20^ which found an effect size of SMD 0.44 (95% CIs 0.12–0.76). In a subgroup analysis in the review by Finch et al, the authors found that much of the effect was observed in trials that were less pragmatic in nature (i.e., more tightly controlled, delivered in a research context), whereas pragmatic trials (i.e., those delivered in more real‐world contexts) demonstrated no significant effect. It is hypothesized that the smaller effect sizes reflected in this review may be due to an increase in the number of trials that are more pragmatic and reflect the real‐world delivery context more closely, and as such could provide a closer estimation of the real‐world impact of ECEC‐based physical activity interventions. As the transition from less pragmatic (e.g. efficacy trials) to more pragmatic (e.g. effectiveness and implementation trials) exists on a continuum,^131^ future reviews may benefit from employing a comprehensive approach to classifying study pragmatism (e.g. PRagmatic Explanatory Continuum Indicator Summary‐2),^132^ to confirm this hypothesis. Nonetheless, findings here continue to reinforce the benefits of interventions delivered within ECEC services on child physical activity. Given such services reach up to 90% of young children,^13^ a benefit of the magnitude found here could significantly improve the activity levels of children at a population level.

The subgroup analysis examining the effectiveness of specific intervention components categorized according to guideline recommendations found that providing opportunities for physical activity, offering educator training, promoting the benefits of physical activity in care, creating environments that promote physical activity, and involving parents, resulted in small, yet statistically significant effects in improving child physical activity. These findings are broadly consistent with that reported in an umbrella review of ECEC‐based physical activity interventions,^18^ and are aligned with play‐based learning approaches outlined within sector frameworks that promote learning and socialization through physical play.^133^ Although these intervention components were typically delivered as part of multi‐component interventions, and so the effect of discrete components on child outcomes remains unknown, findings from this review provide some evidence for the specific components of ECEC‐based physical activity interventions that are likely to warrant broad implementation. Similar to a recent umbrella review,^18^ which found a dearth of primary studies examining single intervention components in this setting, only five included studies employed a single component. More single‐component intervention trials are needed to enable future investigations into the effectiveness of single versus multi‐component interventions.

This review identified non‐significant intervention effects on child physical activity in low SES and/or marginalized populations. Although there were only a small number of studies included in the subgroup meta‐analysis that conducted RCTs in these populations (n = 12), it is important to note a substantial number of studies (n = 29) did not report sufficient information to enable classification of SES or marginalization status of participants. In addition to reporting of data to enable classification, it is likely that more tailored intervention approaches are needed to improve the impact of ECEC‐based physical activity interventions in these populations. Of the included studies that described a positive effect of ECEC‐based physical activity interventions in low SES and/or marginalized populations, interventions included deliberate cultural adaptations surrounding family, music, community, social roles, cognitive and environmental considerations,^44^ co‐development and adaptation of intervention by local stakeholders (e.g. local or district government and ECEC service staff),^111^ and tailored, hands‐on intervention delivery and support to educators, parents and children from local health promoters.^123^ Such approaches allow for more flexibility and adaptation of interventions to meet the specific needs of these populations, whilst also providing an opportunity to address local and contextual barriers to child physical activity and additional outcomes that may be a priority (e.g., literacy).

In terms of secondary outcomes, this review found non‐significant effects on child weight and anthropometrics, however, a significant moderate‐sized effect was found for child FMS outcomes (SMD 0.544). This finding is important given such skills are considered the basic building blocks to more complex movement skills^134^ and are associated with a broader range of health, social, and developmental benefits in young children.^135^ Promisingly, the review found that of the 10 studies that reported on adverse events, none of these found any increases in adverse events or injuries resulting from the interventions. As only a small number of included studies reported on child cognitive functioning (n = 4) and child social–emotional wellbeing (n = 6) outcomes, the true impact of ECEC‐based physical activity interventions on these outcomes is unclear. Finally, while eight studies reported on the cost of the physical activity intervention (or a component of), none of the studies reported on formal economic evaluations of the interventions. As such, the cost‐effectiveness of such interventions is unknown.

### 9.1 Strengths and limitations

A number of strengths of the review are notable. Firstly, the comprehensive electronic database search of eligible trials using a validated search filter, supplemented by searches of gray literature, trial registries, protocols, and author contact was undertaken. While the inclusion of additional databases (e.g. Web of Science and PubMed) may have yielded a greater number of articles, and therefore be recommended for future reviews, it is unlikely that many published RCTs evaluating the impact of ECEC‐based physical activity interventions on child physical activity have been missed in this review. Secondly, the review was limited to studies employing RCT designs given they are considered the gold standard for identifying causation.^21^ Thirdly, the primary outcome of physical activity was restricted to objective measures, increasing the reliability and validity of meta‐analysis findings. Whilst the included study designs and outcome measures may be seen as strengths of the review, such restrictions may also have contributed to the lack of included studies conducted with low SES and/or marginalized populations and therefore may be viewed as limitations.

A potential limitation of the review may include the selection of outcomes measured immediately following intervention delivery. Although this time point was selected to provide an estimation of the most immediate effect of the intervention, the longer‐term (≥12 months) impact of such interventions cannot be determined from this review. As only a few studies reported data according to child sex, we did not undertake subgroup analyses to determine any differential effectiveness of the interventions. Further, similar to previous reviews we did not account for individual study differences in accelerometer or pedometer use, data captured over 24 hours vs in care only, nor individual study data processing decisions, wear time, or cut points.^10^, ^20^ To answer the primary question of the effectiveness of childcare‐based physical activity interventions, the meta‐analysis included only aggregate data synthesis based on average effects between groups and using cut points determined by the study authors, consistent with best practice recommendations.^24^ To allow for standardization of data processes across studies, future reviews undertaking an Individual Participant Data (IPD) approach may be worthwhile.^136^ Finally, as studies not published in English were excluded, it is possible a number of relevant studies have not been included here.

### 9.2 Implications for policy, practice, and research

Findings from this comprehensive review of RCTs of ECEC‐based physical activity interventions support the positive effects of interventions delivered in this setting on child physical activity and FMS. It reinforces the importance of continued investment in these settings from both government and non‐government organizations as crucial to support population‐wide improvements to child physical health and development. Although most interventions included were multicomponent in nature, the review identified specific components of physical activity interventions that were effective in improving child health outcomes, and should therefore be implemented into everyday practice in ECEC services, in particular providing opportunities for children to be physically active, offering educator training, educators promoting the benefits of physical activity, creating an environment that promotes physical activity, and parental involvement. Studies examining the prevalence of implementation of such practices nationally and internationally, however, indicate variable rates ranging between 15% and 98%.^137^, ^138^, ^139^ This evidence‐practice gap suggests that more support for ECEC services to adopt such interventions is required. Given the limited evidence base identifying effective strategies for the implementation of physical activity interventions within ECEC settings,^140^ the methods used to support implementation should undergo careful selection by practitioners to ensure any barriers are addressed to maximize successful implementation.^141^

The intervention components found to be effective in improving child physical activity and FMS also align with international guideline recommendations,^14^ and current learning frameworks for the sector (e.g. The Early Years Learning Framework for Australia).^133^ As such, the inclusion of the delivery of these interventions into local, state, and national policies, and accreditation standards for the sector should be considered.

The review findings reveal substantial implications for research as there are a number of areas where limited evidence exists. Despite the large number of RCTs, there continue to be few RCTs conducted in low SES and/or marginalized populations, targeting children under 2 years of age, and reporting longer‐term effects of ECEC‐based physical activity interventions. Given the importance of this setting as a way of reaching a substantial proportion of young children and parents of differing SES, cultural, and racial backgrounds, more targeted research in these groups is required. Future studies should also include an examination of outcomes ≥12 months post‐intervention to increase our understanding of the longer‐term impacts of such interventions.

There were also few studies that measured the impacts of ECEC‐based physical activity interventions on outcomes identified in the core outcome set for obesity prevention in the early years.^22^ Future studies should measure a broader range of outcomes including social–emotional wellbeing and cognitive functioning to better understand the potential co‐benefits of physical activity interventions. Finally, formal cost‐effectiveness evaluations to understand the economic implications of ECEC‐based physical activity interventions are needed to inform policy decisions and resource allocation in the setting.

## 10 CONCLUSIONS

This systematic review and meta‐analysis provide robust evidence that ECEC‐based physical activity interventions have a positive and significant impact on child physical activity and FMS. While the effects of ECEC‐based physical activity interventions on other outcomes are small, the overall findings emphasize the potential of ECEC interventions to contribute to children's health and wellbeing. Opportunities for future research, particularly around identifying effective interventions for low SES and/or marginalized populations, and understanding the cost‐effectiveness of these interventions, are evident.

## CONFLICT OF INTEREST STATEMENT

Some reviewers are authors of studies likely to be included or included in the review. Authors of potentially relevant studies were not involved in screening, adjudication, data extraction, or risk of bias for studies in which they are involved.

## TRIAL REGISTRATION

The systematic review and meta‐analysis protocol was registered with OSF Registries on September 30, 2022. Identifier: osf‐registrations‐vy3nb‐v1. Protocol can be accessed at: https://osf.io/vy3nb.

## Supporting information


**Table S1:** ECEC physical activity guideline recommendation, practices, and example intervention component descriptions.
Supporting information S2: Search strategy.
Table S3: Characteristics of Included Studies.
Table S4: PRISMA checklist.

## Data Availability

The datasets generated and analyzed during the present study are available from the corresponding author upon reasonable request.
