# Supplementary material for: The impact of early childhood education and care‐based interventions on child physical activity, anthropometrics, fundamental movement skills, cognitive functioning, and social–emotional wellbeing: A systematic review and meta‐analysis
Source: Obes Rev. 2024 Nov 7;26(2):e13852. doi: 10.1111/obr.13852 (PMC11711080; doi:10.1111/obr.13852)
Supplement: Supplementary file 1 — Table S1: ECEC physical activity guideline recommendation, practices, and example intervention component descriptions. Supporting information S2: Search strategy. Table S3: Characteristics of Included Studies. Table S4: PRISMA checklist. [file OBR-26-e13852-s001.pdf]

**Supporting Information**

# The impact of early childhood education and care-based interventions on child physical activity, anthropometrics, fundamental movement skills, cognitive functioning and social-emotional wellbeing: A systematic review and meta-analysis

Alice Grady [1-4] *, Rebecca Lorch [1, 3], Luke Giles [2], Hannah Lamont [2], Amy Anderson [1,3,4], Nicole Pearson [1-3], Maria Romiti [2], Melanie Lum [1-5], Ashleigh Stuart [6], Lucy Leigh [6], Sze Lin Yoong [4,5]

Affiliations:

1. School of Medicine and Public Health, University of Newcastle, Newcastle NSW, Australia
2. Hunter New England Population Health, Hunter New England Local Health District, Newcastle NSW, Australia
3. Population Health Research Group, Hunter Medical Research Institute, New Lambton Heights, NSW, Australia
4. National Centre of Implementation Science (NCOIS), University of Newcastle, Newcastle NSW, Australia
5. Global Centre for Preventive Health and Nutrition, Institute for Health Transformation, Deakin University, Geelong VIC, Australia
6. Data Sciences, Hunter Medical Research Institute, New Lambton Heights, NSW, Australia

***Corresponding author:** Alice Grady, Booth Building, Longworth Avenue, Wallsend NSW 2287, Australia. alice.grady@newcastle.edu.au 02 4924 6310

Table S1: ECEC physical activity guideline recommendation, practices, and example intervention component descriptions

| **ECEC physical activity guideline recommendation** [1] | **ECEC physical activity guideline recommended practices** [1] | **Example intervention component description** |
|---|---|---|
| Provide opportunities for children to be physically active | • Ensure physical activity is incorporated into daily routines and formal childcare curriculum<br>• Include at least 180 min of physical activity of any intensity, spread throughout the day<br>• For children 3–4 years, include at least 60 min of moderate-to-vigorous physical activity each day<br>• Include opportunities for adult-led, structured physical activity<br>• Include opportunities for unstructured physical activity, free play<br>• Provide daily opportunities for activity through outdoor play<br>• Provide opportunities for children to develop and practice gross motor and movement skills<br>• Include culturally appropriate physical activities | • Specific goals for planned, educator-led physical activity opportunities in the classroom (e.g., dancing, obstacle courses); educator-led and child-led physical activity opportunities in outdoor learning spaces (e.g., races, follow the leader); and physical activity integrated into learning opportunities (e.g., acting out stories, counting with large movements/gross motor skills) [2]<br>• Scheduling multiple opportunities for outdoor free-play across the day, every day [3]<br>• Fundamental movement skills sessions over 12 weeks including specific skill instruction and practice [4] |
| Develop and adopt policies for physical activity and physical activity education programs | • Engage staff and parent support for physical activity standards<br>• Seek consultation from experts annually on the physical activity programs delivered<br>• Develop a written policy promoting physical activity and removal of barriers to participation | • Implementation of an evidence-based, stakeholder-informed, written physical activity and sedentary time policy... encompassed eight statements targeting physical activity, outdoor play, and sedentary/screen time [5]<br>• Physical activity program components synonymous with physical activity behaviours such as policy development [6] |
| Offer educator training to provide safe and developmentally appropriate physical activity | • Staff should be trained to provide guidance to parents to encourage physical activity<br>• Staff should be trained to provide guidance to parents in appropriate sleep duration<br>• Staff should be trained in encouraging child physical activity and decreasing sedentary behaviour | • Two training sessions focused on motor skills of children and monthly physical activity tips for educators [7]<br>• A train-the-trainer approach consisting of an experienced master trainer who provided two 3-hour training workshops to ECEC providers and 12 x 1- hour, biweekly "booster" sessions to educators [8] |

| | | |
|---|---|---|
| | • Offer staff annual training opportunities in physical activity programs and practices | |
| Educators to promote the benefits of physical activity with children | • Educators should model and participate in physical activity<br>• Engage children in physical activity they enjoy, including games and sport (age appropriate, fun and variety)<br>• Encourage expressive play e.g. music, dancing, make believe<br>• Educators embed physical activity into educational activities<br>• Avoid punishing children for being physically active<br>• Avoid withholding physical activity as a punishment<br>• Avoid elimination and competitive games<br>• Engage equal participation from boys and girls<br>• Celebrate special occasions with physical activity | • Staff role modelling of active play and delivery of instructional practices (prompts to extend active play and positive statements about children's activity) [9]<br>• Physical activity guide and activity bag containing child-tested activities and materials developed to support educators in providing opportunities for increasing physical activity and improving physical literacy [10]<br>• Opportunities for physical activity were integrated into all aspects of the preschool curriculum, including math, social studies and science, language arts, and nutrition education [11] |
| Limit the time children spend sitting (less is best) | • Children should not be sitting/restrained for more than 30–60 min at a time<br>• When sedentary, children should be engaged in educational, creative and social pursuits<br>• Engage children that tend to be sedentary in active play | • Classroom activity guides included detailed instructions on the delivery of physical activity and sedentary behaviour sessions, and the setup of the classroom environment to encourage physical activity, active play and to reduce sedentary behaviour [12]<br>• Physical activity 'power' breaks with the aim of breaking up sedentary time [6] |
| Limit use of screen time (less is best) | • No screen time for children <2 years<br>• No more than 1 h of screen time/week for children aged ≥2<br>• Screens should not be used during meal or nap times<br>• Limit the use of screen time for educational activities or active movement programs<br>• Parent permission should be requested for children to participate in screen-based activity<br>• Screen time should be supervised by an adult<br>• When offered, screen/digital media should be free from advertising, violence or that tempt overuse<br>• Work with parents to limit overall screen time | • Staff limited the amount of time children spent watching or using electronic media in care [9]<br>• Families encouraged to seek opportunities to reduce the time spent watching television [13] |

| | | |
|---|---|---|
| Support healthy sleeping habits | • Include a nap within the daily routine, with regular sleep and wake-up times<br>• Provide an environment that provides restful sleep: remove screen media and low noise<br>• Maintain a calm nap-time routine | • Sleep curriculum (10 min, 1 afternoon per week) focused on teaching the children the benefits of sleeping and napping [14]<br>• Parents set goals for change and plan actions to meet the goals in a range of areas including sleeping [15] |
| Create an environment that promotes physical activity | • Provide play equipment that encourages physical activity<br>• Provide simple play equipment to encourage creativity and exploration (e.g. cardboard boxes) and portable equipment<br>• Provide adequate space for children to be physically active<br>• Ensure the outdoor area offers variety in terms of secure equipment in shade, open grass and surfaces<br>• Ensure the educator to child ratio is fairly low | • Funding for the rearrangement of the ECEC environment to be more activity-friendly via an indoor movement space and provision of portable or/and fixed indoor or outdoor equipment [16]<br>• Playground equipment such as a sandbox, jungle-gyms (bars for climbing), swing sets, a seesaw, balls, and tricycles [17]<br>• Provision of novel portable play equipment including balls, hula hoops, a hop-scotch mat, obstacle course, stepping domes, ribbon wands, and hop-along bouncers [18] |
| Involve parents in the promotion of physical activity (including reduction of screen time and sedentary behaviours) | • Provide parents education at least 2 times a year<br>• Offer physical activity education to all families in the form of parent/family workshops or meetings | • Stimulus materials and homework activities for families to learn about physical activity, and opportunities for parents/caregivers to participate in the same activities at home that children participated in within care [19]<br>• Children received physical activity homework cards at least once per week; parents invited to three interactive lectures providing information and exchange on healthy development and promotion of motor skills in childhood [20]<br>• Two online webinars for parents to facilitate increased physical activity and hard copies of the physical activity program training materials [21] |

Supporting information S2: Search strategy

Database(s): **Ovid MEDLINE(R) and Epub Ahead of Print, In-Process, In-Data-Review & Other Non-Indexed Citations and Daily** 1946 to October, 2022

Search Strategy:

| # | Searches |
|---|---|
| 1 | randomized controlled trial/ |
| 2 | controlled clinical trial/ |
| 3 | Random Allocation/ |
| 4 | Double-Blind Method/ |
| 5 | single-blind method/ |
| 6 | Placebos/ |
| 7 | *Research Design/ |
| 8 | evaluation studies/ |
| 9 | Comparative Study/ |
| 10 | exp Longitudinal Studies/ |
| 11 | cross-over studies/ |
| 12 | clinical trial.tw. |
| 13 | clinical trial/ |
| 14 | latin square.tw. |
| 15 | (time adj series).tw. |
| 16 | (before adj2 after adj3 (stud* or trial* or design*)).tw. |
| 17 | ((singl* or doubl* or trebl* or tripl*) adj5 (blind* or mask)).tw. |
| 18 | placebo*.tw. |
| 19 | random*.tw. |
| 20 | (matched communities or matched schools or matched populations).tw. |
| 21 | control*.tw. |
| 22 | (comparison group* or intervention stud*).tw. |
| 23 | matched pairs.tw. |
| 24 | (outcome study or outcome studies).tw. |
| 25 | (quasiexperimental or quasi experimental or pseudo experimental).tw. |
| 26 | (nonrandomi?ed or non randomi?ed or pseudo randomi?sed or quasi randomi?ed).tw. |
| 27 | prospectiv*.tw. |
| 28 | volunteer*.tw. |
| 29 | or/1-28 |

| | |
|---|---|
| 30 | exp exercise/ |
| 31 | physical inactivity.mp. |
| 32 | physical activity.mp. |
| 33 | Movement skills.mp. |
| 34 | Motor skills/ |
| 35 | exp Motor Activity/ |
| 36 | (physical education and training).mp. |
| 37 | "Physical Education and Training"/ |
| 38 | exp physical fitness/ |
| 39 | sedentary.tw. |
| 40 | exp life style/ |
| 41 | exp leisure activities/ |
| 42 | exp sports/ |
| 43 | dancing/ |
| 44 | dancing.mp. |
| 45 | (exercise* adj aerobic*).tw. |
| 46 | sport*.tw. |
| 47 | (("lifestyle" or life-style) adj5 activ*).tw. |
| 48 | or/30-47 |
| 49 | pre-school*.tw. |
| 50 | preschool*.tw. |
| 51 | Child Day Care Centers/ |
| 52 | childcare.tw. |
| 53 | child care.tw. |
| 54 | day care.tw. |
| 55 | daycare.tw. |
| 56 | early child*.tw. |
| 57 | (nursery or nurseries).tw. |
| 58 | Kinder*.tw. |
| 59 | or/49-58 |
| 60 | exp Health Education/ |
| 61 | Organizational Policy/ |
| 62 | Public Policy/ |
| 63 | exp Health Policy/ |

| | |
|---|---|
| 64 | exp Inservice Training/ |
| 65 | promot*.tw. |
| 66 | educat*.tw. |
| 67 | program*.tw. |
| 68 | prevention*.tw. |
| 69 | (policy or policies).tw. |
| 70 | train*.tw. |
| 71 | (physical activit* adj6 intervention*).tw. |
| 72 | (physical inactivit* adj6 intervention*).tw. |
| 73 | or/60-72 |
| 74 | 29 and 48 and 59 and 73 |
| **75** | **limit 74 to ed=20140901-20220304** |

Database(s): **Embase** 1947 to present
Search Strategy:

| # | Searches |
|---|---|
| 1 | randomized controlled trial/ |
| 2 | controlled clinical trial/ |
| 3 | randomization/ |
| 4 | double blind procedure/ |
| 5 | single blind procedure/ |
| 6 | placebo/ |
| 7 | Research Design.mp. or *methodology/ |
| 8 | intervention study/ |
| 9 | evaluation study/ |
| 10 | comparative study/ |
| 11 | longitudinal study/ |
| 12 | crossover procedure/ |
| 13 | clinical trial.tw. |
| 14 | clinical trial/ |
| 15 | latin square.tw. |
| 16 | (time adj series).tw. |
| 17 | (before adj2 after adj3 (stud* or trial* or design*)).tw. |
| 18 | ((singl* or doubl* or trebl* or tripl*) adj5 (blind* or mask)).tw. |
| 19 | placebo*.tw. |
| 20 | random*.tw. |
| 21 | (matched communities or matched schools or matched populations).tw. |
| 22 | control*.tw. |
| 23 | comparison group*.tw. |
| 24 | matched pairs.tw. |
| 25 | (outcome study or outcome studies).tw. |
| 26 | (quasiexperimental or quasi experimental or pseudo experimental).tw. |
| 27 | (nonrandomi?ed or non randomi?ed or pseudo randomi?sed or quasi randomi?ed).tw. |
| 28 | prospectiv*.tw. |
| 29 | volunteer*.tw. |
| 30 | or/1-29 |
| 31 | exp exercise/ |

| | |
|---|---|
| 32 | physical inactivit*.mp. |
| 33 | physical activity/ |
| 34 | Movement skills.mp. |
| 35 | Motor skills.mp. or motor performance/ |
| 36 | exp motor activity/ |
| 37 | "physical education and training".mp. |
| 38 | physical education/ |
| 39 | physical fitness.mp. or fitness/ |
| 40 | sedentary lifestyle/ or sedentary.tw. |
| 41 | lifestyle/ |
| 42 | Leisure Activit*.tw. or leisure/ |
| 43 | exp sport/ |
| 44 | dancing/ |
| 45 | dancing.tw. |
| 46 | (exercise* adj aerobic*).tw. |
| 47 | sport*.tw. |
| 48 | (("lifestyle" or life-style) adj5 activ$).tw. |
| 49 | or/31-48 |
| 50 | pre-school*.tw. |
| 51 | preschool*.tw. |
| 52 | day care/ |
| 53 | childcare.tw. |
| 54 | child care.tw. |
| 55 | day care.tw. |
| 56 | daycare.tw. |
| 57 | early child*.tw. |
| 58 | (nursery or nurseries).tw. |
| 59 | Kinder*.tw. |
| 60 | or/50-59 |
| 61 | exp health education/ |
| 62 | health promotion/ |
| 63 | ("organizational policy" or "organisational policy").tw. |
| 64 | Public Policy.mp. |
| 65 | exp health care policy/ |

| | |
|---|---|
| 66 | in service training/ |
| 67 | promot*.tw. |
| 68 | educat*.tw. |
| 69 | program*.tw. |
| 70 | prevention*.tw. |
| 71 | (policy or policies).tw. |
| 72 | train*.tw. |
| 73 | (physical activit* adj6 intervention*).tw. |
| 74 | (physical inactivit* adj6 intervention*).tw. |
| 75 | or/61-74 |
| 76 | 30 and 49 and 60 and 75 |
| **77** | **limit 76 to dd=20140901-20220304** |

Database(s): **APA PsycInfo** 1806 to October 2022
Search Strategy:

| # | Searches |
|---|---|
| 1 | randomized controlled trial.mp. |
| 2 | controlled clinical trial.mp. |
| 3 | Random Allocation.mp. |
| 4 | ("double blind method" or "double blind procedure").mp. [mp=title, abstract, heading word, table of contents, key concepts, original title, tests & measures, mesh word] |
| 5 | ("single blind method" or "single blind procedure").mp. [mp=title, abstract, heading word, table of contents, key concepts, original title, tests & measures, mesh word] |
| 6 | exp Placebo/ |
| 7 | Research Design.mp. or Experimental Design/ |
| 8 | "intervention stud*".mp. |
| 9 | evaluation stud*.mp. |
| 10 | Comparative Stud*.mp. |
| 11 | exp Longitudinal Studies/ |
| 12 | cross-over stud*.mp. |
| 13 | clinical trial.tw. |
| 14 | Clinical Trials/ |
| 15 | latin square.tw. |
| 16 | (time adj series).tw. |
| 17 | (before adj2 after adj3 (stud$ or trial* or design*)).tw. |
| 18 | ((singl* or doubl* or trebl* or tripl*) adj5 (blind* or mask)).tw. |
| 19 | placebo*.tw. |
| 20 | random*.tw. |
| 21 | (matched communities or matched schools or matched populations).tw. |
| 22 | control*.tw. |
| 23 | (comparison group* or control group*).tw. |
| 24 | matched pairs.tw. |
| 25 | (outcome study or outcome studies).tw. |
| 26 | (quasiexperimental or quasi experimental or pseudo experimental).tw. |
| 27 | (nonrandomi?ed or non randomi?ed or pseudo randomi?sed or quasi randomi?ed).tw. |
| 28 | prospectiv*.tw. |
| 29 | volunteer*.tw. |

| | |
|---|---|
| 30 | or/1-29 |
| 31 | exp Exercise/ |
| 32 | physical inactivit*.mp. |
| 33 | exp Physical Activity/ |
| 34 | Movement skills.mp. |
| 35 | exp Motor Skills/ |
| 36 | Motor Activity.mp. |
| 37 | "physical education and training".mp. |
| 38 | exp Physical Education/ |
| 39 | exp Physical Fitness/ |
| 40 | sedentary.mp. |
| 41 | exp Lifestyle/ |
| 42 | exp Recreation/ or exp Leisure Time/ or leisure activities.mp. |
| 43 | exp Sports/ |
| 44 | exp Dance/ |
| 45 | dancing.mp. |
| 46 | (exercise* adj aerobic*).tw. |
| 47 | sport*.tw. |
| 48 | (("lifestyle" or life-style) adj5 activ*).tw. |
| 49 | or/31-48 |
| 50 | exp Preschool Education/ or exp Preschool Students/ or pre-school.mp. |
| 51 | preschool*.mp. |
| 52 | exp Child Day Care/ or exp Day Care Centers/ |
| 53 | exp Child Care/ or childcare.mp. |
| 54 | child care.mp. |
| 55 | day care.tw. |
| 56 | daycare.tw. |
| 57 | early child*.tw. |
| 58 | exp Nursery Schools/ or exp Nursery School Students/ |
| 59 | exp Kindergarten Students/ or Kinder*.mp. |
| 60 | or/50-59 |
| 61 | exp Health Education/ |
| 62 | exp Health Promotion/ |
| 63 | (Organizational Polic* or organisational polic*).mp. |

| | |
|---|---|
| 64 | Public Policy.mp. or exp Government Policy Making/ |
| 65 | exp Health Care Policy/ or Health Policy.mp. |
| 66 | exp Inservice Training/ |
| 67 | promot*.tw. |
| 68 | educat*.tw. |
| 69 | program*.tw. |
| 70 | prevention*.tw. |
| 71 | (policy or policies).tw. |
| 72 | train*.tw. |
| 73 | (physical activit* adj6 intervention*).tw. |
| 74 | (physical inactivit* adj6 intervention*).tw. |
| 75 | or/61-74 |
| 76 | 30 and 49 and 60 and 75 |
| **77** | **limit 76 to up=20140901-20220304** |

**CINAHL**

| # | Query |
|---|---|
| S1 | (MH "Randomized Controlled Trials") |
| S2 | (MH "Clinical Trials+") |
| S3 | (MH "Random Assignment") |
| S4 | (MH "Double-Blind Studies") |
| S5 | (MH "Single-Blind Studies") |
| S6 | (MH "Placebos") |
| S7 | (MH "Study Design") OR "Research Design" |
| S8 | (MH "Experimental Studies") OR "intervention studies" |
| S9 | (MH "Evaluation Research") OR "evaluation studies" |
| S10 | (MH "Comparative Studies") |
| S11 | (MH "Prospective Studies+") OR "Longitudinal Studies" |
| S12 | TI "cross-over stud*" OR AB "cross-over stud*" |
| S13 | TI "clinical trial*" OR AB "clinical trial*" |
| S14 | TI "latin square" OR AB "latin square" |
| S15 | TI (time n1 series) OR AB (time n1 series) |
| S16 | (before n2 after n3 (stud* or trial* or design*)) |
| S17 | ((singl* or doubl* or trebl* or tripl*) n5 (blind* or mask))$ or mask)). |
| S18 | TI placebo* OR AB placebo* |
| S19 | TI random* OR AB random* |
| S20 | ("matched communit*" or "matched school*" or "matched population*") |

| | |
|---|---|
| S21 | TI control* OR AB control* |
| S22 | ("comparison group*" or "control group*") |
| S23 | TI "matched pairs" OR AB "matched pairs" |
| S24 | ("outcome study" or "outcome studies") |
| S25 | (quasiexperimental or "quasi experimental" or "pseudo experimental") |
| S26 | (nonrandomi?ed or "non randomi?ed" or "pseudo randomi?sed" or "quasi randomi?ed") |
| S27 | TI prospective* OR AB prospective* |
| S28 | TI volunteer* OR AB volunteer* |
| S29 | S1 OR S2 OR S3 OR S4 OR S5 OR S6 OR S7 OR S8 OR S9 OR S10 OR S11 OR S12 OR S13 OR S14 OR S15 OR S16 OR S17 OR S18 OR S19 OR S20 OR S21 OR S22 OR S23 OR S24 OR S25 OR S26 OR S27 OR S28 |
| S30 | (MH "Exercise+") |
| S31 | TI "physical inactivit*" OR AB "physical inactivit*" |
| S32 | (MH "Physical Activity") |
| S33 | TI "Movement skills" OR AB "Movement skills" |
| S34 | (MH "Motor Skills") |
| S35 | (MH "Motor Activity") |
| S36 | "physical education and training" |
| S37 | (MH "Physical Education and Training") |
| S38 | (MH "Physical Fitness+") |
| S39 | (MH "Life Style, Sedentary") OR "sedentary" |
| S40 | (MH "Life Style+") |
| S41 | (MH "Leisure Activities+") |

| | |
|---|---|
| S42 | (MH "Sports+") |
| S43 | (MH "Dancing+") |
| S44 | TI dancing OR AB dancing |
| S45 | (exercise* n1 aerobic*) |
| S46 | TI sport* OR AB sport* |
| S47 | (("lifestyle" or life-style) n5 activ*) |
| S48 | S30 OR S31 OR S32 OR S33 OR S34 OR S35 OR S36 OR S37 OR S38 OR S39 OR S40 OR S41 OR S42 OR S43 OR S44 OR S45 OR S46 OR S47 |
| S49 | TI preschool* OR AB preschool* |
| S50 | TI "pre-school*" OR AB "pre-school*" |
| S51 | (MH "Child Day Care") OR "Child Day Care Centers" |
| S52 | TI childcare* OR AB childcare* |
| S53 | TI "child care*" OR AB "child care*" |
| S54 | TI "day care" OR AB "day care" |
| S55 | TI daycare OR AB daycare |
| S56 | TI "early child*" OR AB "early child*" |
| S57 | (MH "Schools, Nursery") OR nursery or nurseries |
| S58 | TI kinder* OR AB kinder* |
| S59 | S49 OR S50 OR S51 OR S52 OR S53 OR S54 OR S55 OR S56 OR S57 OR S58 |
| S60 | (MH "Health Education") OR "Health Education" |
| S61 | (MH "Health Promotion") OR "Health Promotion" |
| S62 | (MH "Organizational Policies") OR (MH "School Policies") |
| S63 | (MH "Public Policy") |

| | |
|---|---|
| S64 | (MH "Health Policy+") |
| S65 | TI "inservice training" OR AB "inservice training" |
| S66 | TI promot* OR AB promot* |
| S67 | TI prevention* OR AB prevention* |
| S68 | TI (policy or policies) OR AB (policy or policies) |
| S69 | TI train* OR AB train* |
| S70 | ("physical activit*" n6 intervention*) |
| S71 | ("physical inactivit*" n6 intervention*) |
| S72 | TI educat* OR AB educat* |
| S73 | TI program* OR AB program* |
| S74 | S60 OR S61 OR S62 OR S63 OR S64 OR S65 OR S66 OR S67 OR S68 OR S69 OR S70 OR S71 OR S72 OR S73 |
| **S75** | **S29 AND S48 AND S59 AND S74 limited September 2014-October 2022** |

## COCHRANE LIBRARY

ID Search Hits

#1 'Exercis* or "physical* inactive*" or "physical* activit*" or "Movement skill*" or "Motor skill*" or "Motor Activ*" or "physical education" or "physical fitness" or sedentary or "life style" or lifestyle or leisure or sport* or danc*

#2 "pre school*" or preschool* or childcare or "child care" or daycare or "day care" or "early child*" or nursery or nurseries or kinder*

#3 "health education" or "health promotion" or policy or policies or promot* or educat* or program* or prevention* or train* or ("physical activity*" and intervention*) or ("physical inactivity*" and intervention*)

#4 AND #1, #2, #3

## ERIC

(Random* or “clinical trial*” or placebo* or “research design*” or “intervention stud*” or “evaluation stud*” or “comparative stud*” or “longitudinal stud*” or “cross over stud*” or “latin square” or “time series” or (before near/2 after near/3 (stud* or trial* or design*)) or ((singl* or doubl* or trebl* or tripl*) near/5 (blind* or mask*)) or “matched communities” or “matched schools” or “matched populations” or control* or “comparison group*” or “control group*” or “matched pairs” or “outcome stud*” or quasiexperimental or “quasi experimental” or “pseudo experimental” or nonrandomi* or “non randomi*” or “pseudo randomi*” or “quasi randomi*” or prospective* or volunteer*) AND (Exercis* or “physical* inactiv*” or “physical* activ*” or “Movement skill*” or “Motor skill*” or “Motor Activ*” or “physical education” or “physical fitness” or sedentary or “life style” or lifestyle or leisure or sport* or danc*) AND (“pre school*” or preschool* or childcare or “child care” or daycare or “day care” or “early child*” or nursery or nurseries or kinder*) AND (“health education” or “health promotion” or policy or policies or promot* or educat* or program* or prevention* or train* or (“physical activity*” w/6 intervention*) or (“physical inactivity*” near/6 intervention*)) Separate searches in abstract, title, subject, identifier fields, then de-duplicated

## Dissertations and Theses

(Random* or “clinical trial*” or placebo* or “research design*” or “intervention stud*” or “evaluation stud*” or “comparative stud*” or “longitudinal stud*” or “cross over stud*” or “latin square” or “time series” or (before near/2 after near/3 (stud* or trial* or design*)) or ((singl* or doubl* or trebl* or tripl*) near/5 (blind* or mask*)) or “matched communities” or “matched schools” or “matched populations” or control* or “comparison group*” or “control group*” or “matched pairs” or “outcome stud*” or quasiexperimental or “quasi experimental” or “pseudo experimental” or nonrandomi* or “non randomi*” or “pseudo randomi*” or “quasi randomi*” or prospective* or volunteer*) AND (Exercis* or “physical* inactiv*” or “physical* activ*” or “Movement skill*” or “Motor skill*” or “Motor Activ*” or “physical education” or “physical fitness” or sedentary or “life style” or lifestyle or leisure or sport* or danc*) AND (“pre school*” or preschool* or childcare or “child care” or daycare or “day care” or “early child*” or nursery or nurseries or kinder*) AND (“health education” or “health promotion” or policy or policies or promot* or educat* or program* or prevention* or train* or (“physical activity*” near/6 intervention*) or (“physical inactivity*” near/6 intervention*))

Separate searches in abstract, title, index term (Keyword), subject heading fields, then de-duplicated

## SCOPUS

ABS TITLE ( ( random* OR "clinical trial*" OR placebo* OR "research design*" OR "intervention stud*" OR "evaluation stud*" OR "comparative stud*" OR "longitudinal stud*" OR "cross over stud*" OR "latin square" OR "time series" OR ( before W/2 after W/3 ( stud* OR trial* OR design* ) ) OR ( ( singl* OR doubl* OR trebl* OR tripl* ) W/5 ( blind* OR mask* ) ) OR "matched communities" OR "matched schools" OR "matched populations" OR control* OR "comparison group*" OR "control group*" OR "matched pairs" OR "outcome stud*" OR quasiexperimental OR "quasi experimental" OR "pseudo experimental" OR nonrandomi* OR "non randomi*" OR "pseudo randomi*" OR "quasi randomi*" OR prospective* OR volunteer* ) AND ( exercis* OR "physical* inactiv*" OR "physical* activ*" OR "Movement skill*" OR "Motor skill*" OR "Motor Activ*" OR "physical education" OR "physical fitness" OR sedentary OR "life style" OR lifestyle OR leisure OR sport* OR danc* ) AND ( "pre school*" OR preschool* OR childcare OR "child care" OR daycare OR "day care" OR "early child*" OR nursery OR nurseries OR kinder* ) AND ( "health education" OR "health promotion" OR policy OR policies OR promot* OR educat* OR program* OR prevention* OR train* OR ( "physical activity*" W/6 intervention* ) OR ( "physical inactivity*" W/6 intervention* ) ) )

## SPORTDISCUS

( random* OR "clinical trial*" OR placebo* OR "research design*" OR "intervention stud*" OR "evaluation stud*" OR "comparative stud*" OR "longitudinal stud*" OR "cross over stud*" OR "latin square" OR "time series" OR ( before n2 after n3 ( stud* OR trial* OR design* ) ) OR ( ( singl* OR doubl* OR trebl* OR tripl* ) n5 ( blind* OR mask* ) ) OR "matched communities" OR "matched schools" OR "matched populations" OR control* OR "comparison group*" OR "control group*" OR "matched pairs" OR "outcome stud*" OR quasiexperimental OR "quasi experimental" OR "pseudo experimental" OR nonrandomi* OR "non randomi*" OR "pseudo randomi*" OR "quasi randomi*" OR prospective* OR volunteer* )

AND ( exercis* OR "physical* inactiv*" OR "physical* activ*" OR "Movement skill*" OR "Motor skill*" OR "Motor Activ*" OR "physical education" OR "physical fitness" OR sedentary OR "life style" OR lifestyle OR leisure OR sport* OR danc* )

AND ( "pre school*" OR preschool* OR childcare OR "child care" OR daycare OR "day care" OR "early child*" OR nursery OR nurseries OR kinder* )

AND ( "health education" OR "health promotion" OR policy OR policies OR promot* OR educat* OR program* OR prevention* OR train* OR ( "physical activity*" n6 intervention* ) OR ( "physical inactivity*" n6 intervention* ) ) )

Separate searches in abstract, title, Keywords, subject heading fields, then de-duplicated

Table S3: Characteristics of Included Studies

| Author, Year, Country, design | Aim | Setting, Population | Number of children/services randomised | Intervention - Duration, guideline recommendation classification, modality, delivery personnel, comparator | Outcome type, outcome, measure, time point | Findings |
|---|---|---|---|---|---|---|
| Adamo 2017 [21-23] Canada Cluster RCT | To evaluate the efficacy of an intervention program delivered in licensed childcare settings, with or without the addition of parent-facilitated home PA promotion, to increase preschoolers' overall PA | ECEC + home<br><br>SES NR | Int 1: 59 children; 6 services<br><br>Int 2: 94 children; 6 services<br><br>Con: 62 children; 6 services | 6 months<br><br>Int 1: Provide opportunities for children to be physically active; offer educator training to provide safe and developmentally appropriate PA; educators to promote the benefits of PA with children; create a physical environment that promotes PA<br><br>Int 2: Provide opportunities for children to be physically active; offer educator training to provide safe and developmentally appropriate PA; educators to promote the benefits of PA with children; create a physical environment that promotes PA; parental involvement<br><br>Face to face; written | PA: MVPA (min/h); Accelerometer (Actical); Wear time: 7 days, valid if 5 hours/day for 3 days; Cut points: Adolph et al 2012[24]<br><br>Weight/anthropometrics: BMI; portable digital scale and stadiometer<br><br>FMS: GMQ; TGMD-2<br><br>Wellbeing: HRQoL; PedsQL™ 4.0<br><br>6 months | No significant differences between groups in changes from baseline to follow-up for MVPA (min/h) ($p = 0.32$),<br>BMI ($p = 0.42$), GMQ ($p = 0.49$) or HRQoL ($p = 0.2$) |

| | | | | | | |
|---|---|---|---|---|---|---|
| | | | | Childcare staff; other org/expert<br><br>Waitlist control | | |
| Aivazidis 2019 [25]<br>Greece<br>Cluster RCT | To examine the effects of a multifaceted intervention involving classroom teachers and PE teachers on the motor competence and PA of kindergarten children. | ECEC + home<br><br>SES NR | Int: 74 children; 2 services<br><br>Con: 66 children; 2 services | 8 months<br><br>Provide opportunities for children to be physically active; offer educator training to provide safe and developmentally appropriate PA; create a physical environment that promotes PA; parental involvement<br><br>Face to face<br><br>Research team; childcare staff; other org/expert<br><br>Usual care | PA: Step counts per day; Pedometer (Omron HJ-720IT-E2); Wear time: 5 weekdays, valid if 7 hours/day for 3 days; Cut points: De Craemer et al 2015[26]<br><br>FMS: Jumping sideways; Korper-koordination Test for Kinder (KTK)<br><br>8 months | Significant differences between groups in change from baseline in step counts per day ($p < .001$) and jumping sideways ($p < .001$) |
| Alhassan 2007 [17]<br>USA<br>RCT | To explore the effects of increasing recess time on total daily PA levels in Latino children attending full-day preschool | ECEC<br><br>Low SES and/or marginalised population | Int: 18 children<br><br>Con: 15 children<br><br>1 service | 2 days<br><br>Provide opportunities for children to be physically active; create a physical environment that promotes PA<br><br>Face to face<br><br>Childcare staff | PA: Total daily % time in MVPA; Accelerometer (ActiGraph); Wear time: 5 days, valid if 10 hours/day; Cut points: Sirard et al 2005[27]<br><br>2 days | No significant differences between groups in change from baseline for total daily % time in MVPA ($p > 0.05$) |

| | | | | | | |
|---|---|---|---|---|---|---|
| | | | | Usual care | | |
| Alhassan 2012 [28]<br>USA<br>Cluster RCT | To examine the effect of a classroom teacher-taught, locomotor-based PA program on the LMS and PA levels of minority preschoolers | ECEC<br><br>Low SES and/or marginalised population | Int: 69 children<br><br>Con: 45 children<br><br>2 services, 8 classrooms (Int vs Con NR) | 6 months<br><br>Provide opportunities for children to be physically active; offer educator training to provide safe and developmentally appropriate PA; create a physical environment that promotes PA<br><br>Face to face; written<br><br>Childcare staff; other org/expert<br><br>Usual care | PA: Total daily % time in MVPA; Accelerometer (ActiGraph GT1M); Wear time: 7 days, valid if 9 hours/day for 4 weekdays; Cut points: Sirard et al 2005[27]<br><br>FMS: LMS percentile; TGMD-2<br><br>6 months | No significant differences in groups for changes in total daily % time in MVPA ($p = 0.23$) or LMS percentile ($p = 0.19$) |
| Alhassan 2013 [29]<br>USA<br>Cluster RCT | To examine the effects of an additional structured outdoor PA programme on preschoolers' PA levels | ECEC<br><br>SES NR | Int: 4 classrooms<br><br>Con: 4 classrooms<br><br>2 services; 134 children (Int vs Con NR) | 4 weeks<br><br>Offer educator training to provide safe and developmentally appropriate PA; educators to promote the benefits of PA with children; create a physical environment that promotes PA<br><br>Face to face; written | PA: Total daily % time in MVPA; Accelerometer (ActiGraph GT1M); Wear time: 7 days, valid if 9 hours/day for 4 weekdays; Cut points: Sirard et al 2005[27]<br><br>4 weeks | Significant increase was observed in the intervention group total daily % time spent in MVPA ($p = 0.03$) compared to control |

| | | | | | | |
|---|---|---|---|---|---|---|
| | | | | Research team; childcare staff<br><br>Usual care | | |
| Alhassan 2019 [14]<br>USA<br>Cluster RCT | To examine the feasibility and preliminary efficacy of integrating a 12-week health behaviour intervention into early education learning standards on PA, diet, and sleep (PADS) behaviour of preschoolers. | ECEC + home<br><br>SES NR | Int: 60 children; 1 centre<br><br>Con: 54 children; 1 centre | 12 weeks<br><br>Provide opportunities for children to be physically active; offer educator training to provide safe and developmentally appropriate PA; educators to promote the benefits of PA with children; support healthy sleeping habits; parental involvement<br><br>Face to face; written<br><br>Research team; childcare staff<br><br>Usual care | PA: Total daily % time in MVPA; Accelerometer (ActiGraph GT1M, GT3X, GT3X+, and ActiSleep); Wear time: 7 days, valid if 3 days; Cut points: Pate et al 2006[30]<br><br>Adverse events: number of adverse effects to daily routines; NR<br><br>12 weeks | Significant increase from baseline in the total daily % time in MVPA (p = 0.02) in the intervention compared to control<br><br>No adverse effects were reported |
| Alhassan 2022 [31]<br>USA<br>Cluster RCT | To examine the feasibility and preliminary efficacy of a 10-week program to alter toddlers' time spent in total PA [light to moderate-to-vigorous PA (MVPA)] during the childcare day. | ECEC<br><br>SES NR | Int: 45 children; 2 services<br><br>Con: 27 children; 1 service | 10 weeks<br><br>Provide opportunities for children to be physically active; offer educator training to provide safe and developmentally appropriate PA; educators to promote the benefits of PA with children<br><br>Face to face; written | PA: Linear change of minutes in MVPA; Accelerometer (ActiGraph GT1M, GT3X, and GT3X + BT); Wear time: 4 days; Cut points: Trost et al 2012[11]<br><br>10 weeks | Significant difference between groups linear change of minutes in MVPA (p = 0.032) from baseline |

| | | | | | | |
|---|---|---|---|---|---|---|
| | | | | Research team; childcare staff<br><br>Waitlist control | | |
| Andersen 2020 [32]<br>Norway<br>Cluster RCT | To investigate the effectiveness of the "Active Kindergarten – Active Children" study to increase children's PA levels and reduce sedentary time within the ECEC setting | ECEC<br><br>SES NR | Int: 67 children; 6 services<br><br>Con: 49 children; 5 services | 4 months<br><br>Provide opportunities for children to be physically active; offer educator training to provide safe and developmentally appropriate PA; educators to promote the benefits of PA with children; limit the time children spend sitting; create a physical environment that promotes PA<br><br>Face to face; online; written<br><br>Research team; childcare staff<br><br>Waitlist control | PA: MVPA (mins/day); Accelerometer (ActiGraph GT3X+); Wear time: 5 days, valid if 6 hours/day for 2 days; Cut points: Butte et al 2014 [33]<br><br>4 months | Significant increase in time spent in MVPA by 10 min/day (p = 0.01) in intervention compared to the control |
| Annesi 2013 [34]<br>USA<br>Cluster RCT | To test the hypotheses that a theory-based treatment would be associated with significantly increased vigorous and MVPA, and decreased sedentary time, across the | ECEC<br><br>Low SES and/or marginalised population | Int: 202 children; 11 classrooms<br><br>Con: 136 children; 8 classrooms<br><br>7 services | 8 weeks<br><br>Provide opportunities for children to be physically active; offer educator training to provide safe and developmentally appropriate PA; educators to promote the benefits of PA with children<br><br>Face to face; written | PA: % of time in MVPA; Accelerometer (ActiGraph GT3X); Wear time: 4.75 hours/1 day; Cut points: Pate et al 2006[30]; Pate et al 2004[35]; Sirard et al 2005[27]<br><br>8 weeks | After controlling for age and sex, changes in % of time in MVPA significantly increased in the intervention compared to control (p = 0.026) |

| | | | | | | |
|---|---|---|---|---|---|---|
| | preschool day relative to a control condition of usual care. | | | Childcare staff<br><br>Usual care | | |
| Bellows 2013 [36]<br>USA<br>Cluster RCT | To assess the efficacy of an intervention on gross motor skill performance, physical activity, and weight status of preschoolers. | ECEC + home<br><br>Low SES and/or marginalised population | Int: 132 children; 18 classrooms; 4 services<br><br>Con: 131 children; 13 classrooms; 4 services | 18 weeks<br><br>Provide opportunities for children to be physically active; offer educator training to provide safe and developmentally appropriate PA; create a physical environment that promotes PA; parental involvement<br><br>Face to face; written<br><br>Research team; childcare staff<br><br>12-week nutrition program | PA: Mean steps per day; Pedometer (Walk4Life Classic); Wear time: 6 days; Cut points: N/A<br><br>FMS: GMQ; PDMS-2<br><br>Weight/anthropometrics: BMI z-score; Portable stadiometer, electronic scale, EpiInfo (BMI z-zcore)<br><br>18 weeks | No intervention effect was found for change in mean steps per day (p > 0.05) or BMI z-score from baseline<br><br>Significant changes in GMS (p <0.0005) in the intervention compared to control |
| Bonvin 2013 [16]<br>Switzerland<br>Cluster RCT | To assess the effect of a governmentally led centre based childcare PA program on child motor skill and possible effects on children's body mass index, childcare | ECEC + home<br><br>SES NR | Int: 313 children; 29 services<br><br>Con: 335 children; 29 services | 9 months<br><br>Provide opportunities for children to be physically active; offer educator training to provide safe and developmentally appropriate PA; create a physical environment that promotes PA; parental involvement<br><br>Face to face; written | PA: MVPA (epochs/hour ≥ 420 counts); Accelerometer (ActiGraph GT1M); Wear time: 1 day, valid if 3 hours; Cut points: Pate et al 2006[30]<br><br>FMS: Global motor score; Adapted Zurich Neuromotor Assessment test | No significant changes between groups in MVPA (p = 0.68), motor skills (p = 0.43), BMI (p = 0.29) or QoL (0.46) |

| | | | | | | |
|---|---|---|---|---|---|---|
| | based PA level and QoL | | | Research team; childcare staff; other org/expert<br><br>Usual care | Weight/anthropometrics: BMI; Electronic scale<br><br>Wellbeing: QoL; PedsQL 4.0<br><br>~9 months | |
| Byun 2018 [37]<br>USA<br>Cluster RCT | To evaluate the feasibility and effectiveness of an intervention that employed a technology-based PA monitoring system and teacher-regulated strategies to promote PA in preschoolers. | ECEC<br><br>SES NR | Int: 58 children; 3 services<br><br>Con: 57 children; 2 services | 1 week<br><br>Provide opportunities for children to be physically active; offer educator training to provide safe and developmentally appropriate PA; educators to promote the benefits of physical activity with children; create a physical environment that promotes PA<br><br>Face to face; written<br><br>Research team; childcare staff<br><br>Waitlist control | PA: TPA (minutes); Accelerometer (ActiGraph GT3X+); Wear time: 5 days, valid if 5 hours/day for 3 days; Cut Points: Pate et al 2006[30]<br><br>1 week | Intervention group showed significantly higher levels of TPA than children in the control group (p <0.05) at follow-up |
| Cardon 2009 [38]<br>Belgium<br>Cluster RCT | To investigate the effects of providing play equipment and markings at the preschool | ECEC<br><br>SES NR | Int 1: 161 children; 10 services<br><br>Int 2: 150 children; 10 services | 6 weeks<br><br>Int 1,2,3: Create a physical environment that promotes PA | PA: % of time in MVPA; Accelerometer (ActiGraph GT1M); Wear time: 1 hour before recess to after | The 3 interventions were not effective in increasing the % of time in MVPA from baseline (p >0.05) |

| | | | | | | |
|---|---|---|---|---|---|---|
| | playground on PA levels | | Int 3: 161 children; 10 services<br><br>Con: 162 children; 10 services | Face to face<br><br>Research team; childcare staff<br><br>Usual care | recess; Cut points: Sirard et al 2005[27]<br><br>6 weeks | |
| Carroll 2021 [39]<br>USA<br>Cluster RCT | To examine the effects of an indoor teacher-guided and led preschool PA intervention in low-income schools. | ECEC<br><br>Low SES and/or marginalised population | Int: 35 children; 3 services<br><br>Con: 31 children; 3 services | 3 weeks<br><br>Provide opportunities for children to be physically active<br><br>Face to face<br><br>Research team; childcare staff<br><br>Usual care | PA: MVPA indoor time (mean %); Accelerometer (ActiGraph GT3X); Wear time: 6 days over 3 weeks; Cut points: Butte et al 2014[33]<br><br>3 weeks | Significant group by time interaction in children's MVPA levels during indoor time ($p = 0.036$) |
| Chow 2016 [10]<br>Canada<br>Cluster RCT | To evaluate the impact of a multimodal PA and healthy eating intervention on educators' provision of opportunities for children to improve their PA levels, FMS and healthy eating behaviours. | ECEC<br><br>SES NR | Int: 42 children; 3 services<br><br>Con: 27 children; 3 services | 48 weeks<br><br>Provide opportunities for children to be physically active; offer educator training to provide safe and developmentally appropriate PA; educators to promote the benefits of physical activity with children; create a physical environment that promotes PA<br><br>Face to face; written<br><br>Research team; childcare staff | PA: MVPA (mean weekday mins); Accelerometer (Actical); Wear time: 7 days; Cut points: Puyau et al 2004[40]<br><br>FMS: GMQ; TGMD-2<br><br>48 weeks | Significant between group differences in MVPA levels at follow-up ($p < 0.05$)<br><br>No significant between group differences in TGMD-2 scores at follow-up |

| | | | | | | |
|---|---|---|---|---|---|---|
| | | | | Waitlist control | | |
| De Bock 2013 [41] Germany Cluster RCT | To test whether a participatory parent-focused leads to changes in preschoolers' objectively measured PA and sedentary behaviour, both at home and in school | ECEC + home + community<br><br>SES NR | Int: 534 children; 19 services<br><br>Con: 494 children; 20 services | 9 months<br><br>Provide opportunities for children to be physically active; offer educator training to provide safe and developmentally appropriate PA; educators to promote the benefits of physical activity with children; create a physical environment that promotes PA; parental involvement<br><br>Face to face; online; written; video<br><br>Research team; other org/expert<br><br>Usual care | PA: MVPA (min/day); Accelerometer (Actihart monitors); Wear time: 7 days, valid if 1 weekday and 1 weekend day; Cut points: De Bock et al 2010[42]<br><br>Weight/anthropometrics: BMI; Scales and stadiometer<br><br>Wellbeing: QoL; KINDL-R QoL measure<br><br>9 months | No significant differences between groups in time in MVPA ($p > 0.1$) and mean change of BMI ($p = 0.41$) from baseline<br><br>Significant increase in perceived QoL in the intervention compared to control ($p = 0.007$) |
| De Craemer 2014 [43] Belgium Cluster RCT | To examine the effect of the Toy Box-intervention on increasing Belgian preschoolers' objectively measured PA levels | ECEC + home<br><br>Low, medium + high SES | Int: 696 children; 15 services<br><br>Con: 454 children; 12 services | 24 weeks<br><br>Provide opportunities for children to be physically active; offer educator training to provide safe and developmentally appropriate PA; create a physical | PA: Total daily % time in TPA; Accelerometer (ActiGraph GT1M, GTX3, GTX3+); Wear time: 6 days, valid if 6 hours/day for 2 weekdays and 1 weekend day; Cut | No significant intervention effect was found for % time in TPA, from baseline to follow-up, compared to the control |

| | | | | | | |
|---|---|---|---|---|---|---|
| | | | | environment that promotes PA<br><br>Face to face; written<br><br>Research team; childcare staff<br><br>Usual care | points: Evenson et al 2008[44]<br><br>48 weeks | |
| De Craemer 2017 [45] Belgium, Bulgaria, Germany, Greece, Poland and Spain Cluster RCT | To evaluate the effectiveness of the ToyBox-intervention on European preschoolers' objectively measured steps per day in the total sample and in the country-specific samples | ECEC + home<br><br>Low, medium + high SES | Int: 2040 children; 188 services<br><br>Con: 1012 children; 121 services | 24 weeks<br><br>Provide opportunities for children to be physically active; offer educator training to provide safe and developmentally appropriate PA; educators to promote the benefits of PA with children; create a physical environment that promotes PA; parental involvement<br><br>Face to face; written<br><br>Research team; childcare staff<br><br>Usual care | PA: Steps per average day; Pedometer (Omron HJ-720IT-E2) and accelerometer (ActiGraph); Wear time: 6 days, valid if 2 weekdays and 1 weekend day; Cut points: N/A<br><br>52 weeks | No significant intervention effects on change in children' steps per average day from baseline were found ($p > 0.05$) |
| Driediger 2019 [46] Canada Cluster RCT | In an extension of the Supporting Physical Activity in the Childcare Environment (SPACE) cluster | ECEC<br><br>SES NR | Int: 102 children; 6 services<br><br>Con: 83 children; 6 services | 8 weeks<br><br>Provide opportunities for children to be physically active<br><br>Face to face | PA: Mins/hour of MVPA; Accelerometer (Actical Z and B series); Wear time: 5 days, valid if 5 hours/day for 2 days; | No significant differences in MVPA were observed between groups over time ($p = 0.43$) |

| | | | | | | |
|---|---|---|---|---|---|---|
| | RCT, to explore the impact of four 30-min daily outdoor unstructured play periods on preschoolers MVPA | | | Childcare staff<br><br>Usual care | Cut points: Adolph et al 2012[24]<br><br>8 weeks | |
| Duff 2019 [47] Ireland Cluster RCT | To evaluate the impact of a six-week pilot program on educator confidence, as well as children's PA levels and FMS proficiency | ECEC<br><br>SES NR | Int: 95 children; 5 services<br><br>Con: 100 children; 5 services | 6 weeks<br><br>Provide opportunities for children to be physically active; offer educator training to provide safe and developmentally appropriate PA; educators to promote the benefits of PA with children<br><br>Face to face; written<br><br>Childcare staff<br><br>Waitlist control | PA: Mins of total PA over 3 hour period; Accelerometer (ActiGraph GT1M, GT3X); Wear time: 5 days, valid if 2.5 hours/day for 3 days, Cut points: Espana-Romero et al 2013[48]; Pate et al 2006[30]<br><br>FMS: total FMS score; TGMD-2<br><br>6 weeks | No significant differences between groups for change in children's mins of PA or FMS score (p values NR) |
| Eliakim 2007 [49] Israel Cluster RCT | To study the effects of a brief 14 week school based health promotion intervention on habitual PA and BMI | ECEC<br><br>Upper-middle SES | Int: 54 children; 2 services<br><br>Con: 47 children; 2 services | 4 months<br><br>Provide opportunities for children to be physically active; educators to promote the benefits of PA with children; limit the time children spend sitting; limit the use of screen time<br><br>Face to face | PA: Mean steps/day (during and after school hours); Pedometer (Stepometer); Wear time: 3 days; Cut points: N/A<br><br>Weight/anthropometrics: BMI percentile; Standard calibrated scales and stadiometers | Significant increase from baseline in mean steps per day ($p < 0.003$) and a greater decrease in BMI percentile ($p < 0.05$) in intervention group compared to control<br><br>No adverse events were reported |

| | | | | | | |
|---|---|---|---|---|---|---|
| | | | | Research team; childcare staff; other org/expert<br><br>Usual care | Adverse events: Number of adverse events; NR<br><br>4 months | |
| Ellis 2019 [50] Australia Cluster RCT | To investigate the feasibility, acceptability and potential efficacy of a childcare based intervention to reduce sitting time among pre-schoolers, and to assess whether a reduction in sitting time has an effect on executive function | ECEC<br><br>SES NR | Int: 55 children; 2 services<br><br>Con: 60 children; 2 services | 12 weeks<br><br>Provide opportunities for children to be physically active; offer educator training to provide safe and developmentally appropriate PA; educators to promote the benefits of PA with children; limit the time children spend sitting; create a physical environment that promotes PA<br><br>Face to face; written<br><br>Childcare staff<br><br>Waitlist control | PA: % of time per day spent stepping; Accelerometer (ActivPAL); Wear time: days in care/1 week, valid if 3 hours/day for 1 day; Cut points: N/A<br><br>Weight/anthropometrics: BMI; Stadiometer and electronic scale<br><br>Cognitive functioning: inhibition (executive function); Early Years Toolbox ('Go/No-Go' game)<br><br>12 weeks | No significant difference in change from baseline between groups for % of time spent stepping per day (p = 0.06), BMI (p = 0.34) or inhibition (p = 0.17) |
| Engel 2021 [4] Australia Cluster RCT | To evaluate the effect of the PLAYFun Program on FMS competence and PA levels within a preschool setting | ECEC<br><br>High SES | Int: 22 children; 2 services<br><br>Con: 17 children; 2 services | 12 weeks<br><br>Provide opportunities for children to be physically active; educators to promote the benefits of PA with children | PA: % of time spent in MVPA during care; Accelerometer (ActiGraph GT3X); Wear time: 2 days, valid if 5 hours/day for 2 days Cut points: Butte et al 2014[33] | No significant difference in change from baseline between groups for % of time spent in MVPA during care at follow-up (p = 0.361) |

| | | | | | | |
|---|---|---|---|---|---|---|
| | | | | Face to face<br><br>Research team<br><br>Waitlist control | FMS: total GMQ; TGMD-2<br><br>12 weeks | At follow-up, there was a significant increase in GMQ (p = 0.01) |
| Finch 2014 [9] Australia Cluster RCT | To evaluate the impact of a multilevel intervention on the PA levels of 3- to 5-year-old children attending center-based childcare services | ECEC<br><br>Low + high SES | Int: 242 children; 10 services<br><br>Con: 215 children; 10 services | 4 months<br><br>Provide opportunities for children to be physically active; develop and adopt policies for PA and PA education programs; offer educator training to provide safe and developmentally appropriate PA; educators to promote the benefits of PA with children; limit the time children spend sitting; limit the use of screen time; create a physical environment that promotes PA<br><br>Face to face; telephone; written; DVD<br><br>Research team; childcare staff<br><br>Usual care | PA: Mean step count per minute (during care hours); Pedometer (Yamax SW200, SW700); Wear time: 1 day; Cut points: N/A<br><br>Adverse Events: injury rate per month; NR<br><br>24 weeks | Between-group differences analysis comparing rate of change in mean step counts per minute from baseline to follow-up was non-significant (p = 0.07)<br><br>No significant difference observed in the injury rate per month (p = 0.85) |
| Fitzgibbon 2011 [51] USA Cluster RCT | To assess the feasibility and effectiveness of a teacher-delivered | ECEC + home<br><br>Low SES and/or | Int: 376 children; 9 services | 14 weeks<br><br>Provide opportunities for children to be physically | PA: MVPA (min/day); Accelerometer (ActiGraph GT1M); Wear time: 7 days, valid | At follow-up, children in the intervention group engaged in significantly more MVPA than children in the control (p = 0.02) |

| | | | | | | |
|---|---|---|---|---|---|---|
| | weight control intervention for black preschool children | marginalised population | Con: 353 children; 9 services | active; offer educator training to provide safe and developmentally appropriate PA; limit the use of screen time; parental involvement<br><br>Face to face; written; CD<br><br>Research team; childcare staff<br><br>Health lessons for children; weekly parent newsletter; teacher training; monthly meeting with intervention co-ordinator | if 8 hours/day for 4 days; Cut points: Pate et al 2006[30]<br><br>Weight/anthropometrics:<br>BMI z-score; Digital scale and stadiometer<br><br>14 weeks | There was no significant between group difference in BMI z-score (p = 0.39) |
| Goldfield 2016 [8]<br>Canada<br>Cluster RCT | To evaluate the efficacy of intervening with childcare providers to increase overall PA levels and reduce adiposity in children aged 3–5 years attending licensed childcare services | ECEC<br><br>SES NR | Int: 40 children; 3 services<br><br>Con: 43 children; 3 services | 6 months<br><br>Provide opportunities for children to be physically active; offer educator training to provide safe and developmentally appropriate PA; educators to promote the benefits of PA with children; limit the time children spend sitting; create a physical environment that promotes PA<br><br>Face to face; written<br><br>Childcare staff; other org/expert | PA: MVPA (mins/preschool day); Accelerometer (Actical); Wear time: 5 days, valid if 4 hours/day for 2 days; Cut points: Adolph et al 2012[24]; Pfeiffer et al 2006[52]<br><br>Weight/anthropometrics: BMI z-score; Stadiometer and digital scale<br><br>FMS: GMQ; TGMD-2<br><br>6 months | Groups did not significantly differ in changes in MVPA (p = 0.085) or BMI z-score (p = 0.225)<br><br>Children in the intervention group demonstrated significantly greater improvement in their standardized GMQ (p = 0.025) |

| | | | | | | |
|---|---|---|---|---|---|---|
| | | | | Waitlist control | | |
| Hoffman 2020 [53]<br>USA<br>Cluster RCT | To test the Wellness Enhancing Physical Activity in Young Children (WE PLAY) program, on children's MVPA | ECEC<br><br>Low SES and/or marginalised population | Int: 108 children; 3 services<br><br>Con: 144 children; 3 services | 4 weeks<br><br>Provide opportunities for children to be physically active; offer educator training to provide safe and developmentally appropriate PA; educators to promote the benefits of PA with children;<br><br>Face to face; online; written; video<br><br>Childcare staff; other org/expert<br><br>Usual care | PA: Average mins/hr of MVPA per hour during school hours; Accelerometer (ActiGraph GT9X); Wear time: 5 days, valid if 50% of school hours/day for 3 days; Cut points: Pate et al 2006[30]<br><br>4 weeks | No significant post-test difference at follow-up between groups in MVPA (p = 0.22) |
| Jones 2011 [54]<br>Australia<br>Cluster RCT | To assess the feasibility, acceptability and potential efficacy of a PA programme for preschool children | ECEC<br><br>SES NR | Int: 52 children; 1 centre<br><br>Con: 45 children; 1 centre | 20 weeks<br><br>Provide opportunities for children to be physically active; offer educator training to provide safe and developmentally appropriate PA; educators to promote the benefits of PA with children; create a physical environment that promotes PA<br><br>Face to face | PA: % time in MVPA; Accelerometer (ActiGraph MTI7164); Wear time: 2 days; Cut points: Sirard et al 2005[27]<br><br>FMS: total movement skill development score; TGMD-2<br><br>Weight/anthropometrics: BMI; NR | No significant intervention effects observed in change from baseline for % of time in MVPA (p = 0.73) or BMI (p = 0.53)<br><br>Children in the intervention group showed significantly greater improvements for overall movement skill (p = 0.00) compared to control |

| | | | | | | |
|---|---|---|---|---|---|---|
| | | | | Research team; childcare staff<br><br>Usual care | 24 weeks | |
| Jones 2016 [55]<br>Australia<br>Cluster RCT | To evaluate the implementation of a gross motor skills and PA program (Jump Start) when facilitated solely by childcare educators within their own centre | ECEC<br><br>SES NR | Int: 82 children; 2 services<br><br>Con: 55 children; 2 services | 6 months<br><br>Provide opportunities for children to be physically active; offer educator training to provide safe and developmentally appropriate PA; educators to promote the benefits of PA with children; create a physical environment that promotes PA<br><br>Face to face; written; video<br><br>Childcare staff<br><br>Waitlist control | PA: % of time spent in MVPA during care; Accelerometer (ActiGraph GT3X+); Wear time: 2 days; Cut points: Pate et al 2006[30]<br><br>FMS: GMS total score; TGMD-2<br><br>Adverse events: number of child injuries; NR<br><br>6 months | No statistically significant differences in change from baseline for % of time spent in MVPA (p = 0.43) or GMS total score (p = 0.3) between groups<br><br>No injuries were reported throughout the intervention. |
| Kipping 2019 [15]<br>UK<br>Cluster RCT | To evaluate the feasibility and acceptability of implementing and trialling an adaptation of the NAP SACC intervention, with a home component in nursery settings | ECEC + home<br><br>Low, medium + high SES | Int: 247 children; 6 services<br><br>Con: 229 children; 6 services | 5 months<br><br>Provide opportunities for children to be physically active; develop and adopt policies for PA and PA education programs; offer educator training to provide safe and developmentally appropriate PA; educators to promote the benefits of PA with children; limit the time children spend sitting; limit | PA: MVPA (mins per weekday); Accelerometer (ActiGraph GT1M); Wear time: 5 days, valid if 8 hours/day; Cut points: Evenson et al 2008[44]; Puyau et al 2004[40]<br><br>Weight/anthropometric s: BMI z-score; | The intervention increased MVPA on nursery days compared to control (p value NR)<br><br>At follow-up, there was a greater increase in BMI z-score in the intervention arm compared to control (p value NR)<br><br>There was no change in the total PedsQL score for intervention compared to control (p value NR) |

| | | | | | | |
|---|---|---|---|---|---|---|
| | | | | the use of screen time; support healthy sleeping habits; create a physical environment that promotes PA; parental involvement<br><br>Face to face; telephone; online; written; SMS<br><br>Childcare staff; other org/expert<br><br>Usual care | Stadiometer and digital scale<br><br>Wellbeing: Total PedsQL; PedsQL 4.0 score<br><br>Adverse events: number of adverse events; adverse event/incident report forms<br><br>8-10 months | No adverse events were reported |
| Leis 2020 [56] Canada Cluster RCT | To assess the effectiveness of the HSDS intervention in increasing PA levels and healthy eating as well as improving fundamental movement skills in preschoolers attending ECC | ECEC + home<br><br>SES NR | Int: 464 children; 31 services<br><br>Con: 433 children; 30 services | 6-8 months<br><br>Provide opportunities for children to be physically active; offer educator training to provide safe and developmentally appropriate PA; create a physical environment that promotes PA<br><br>Face to face; telephone; online; written<br><br>Research team; childcare staff; other org/expert<br><br>Usual care | PA: Minutes in MVPA per day; Accelerometer (Actical); Wear time: 5 days, valid if 2 hours/day on 4 days; Cut points: Pfeiffer et al 2006[52]<br><br>FMS: locomotor score; TGMD-2<br><br>9 months | No significant differences in change from baseline between groups in minutes in MVPA per day ($p = 0.3$)<br><br>Children in the intervention group had a significantly greater increase in their locomotor skills scores than children in the control group ($p = 0.001$) |
| Malden 2019 [12] | To test the feasibility of a | ECEC + home | Int: 26 children; 3 services | 18 weeks | PA: Minutes of total daily PA/day; | At follow up, both intervention and control groups had small |

| | | | | | | |
|---|---|---|---|---|---|---|
| UK (Scotland) Cluster RCT | cluster RCT of the ToyBox-Scotland preschool obesity prevention programme to inform the design of a future full-scale RCT | Low SES and/or marginalised population | Con: 16 children; 3 services | Provide opportunities for children to be physically active; offer educator training to provide safe and developmentally appropriate PA; educators to promote the benefits of PA with children; limit the time children spend sitting; limit the use of screen time; create a physical environment that promotes PA; parental involvement<br><br>Face to face; written<br><br>Research team; childcare staff<br><br>Usual care | Accelerometer (ActivPAL3); Wear time: 7 days, valid if 3 days; Cut points: Not reported<br><br>Weight/anthropometrics: BMI z-score; Stadiometer and electronic scale<br><br>15-17 weeks | decreases in PA, with larger decreases in the intervention group, and showed small increases in BMI z-scores, with larger increases in the control group (p values NR) |
| Mavilidi 2021 [57] Greece Cluster RCT | To examine the effect of training early childhood educators to promote PA in preschools on children's PA levels | ECEC<br><br>Low SES and/or marginalised population | Int: 80 children; 4 services<br><br>Con: 70 children; 3 services | 4 weeks<br><br>Offer educator training to provide safe and developmentally appropriate PA; educators to promote the benefits of PA with children<br><br>Face to face; written<br><br>Research team; childcare staff<br><br>Usual care | PA: Mean number of steps per day; Pedometer (Omron HJ-720IT); Wear time: 2 days; Cut points: N/A<br><br>12 weeks | No significant differences between groups in children's number of steps at follow-up (p = 0.76) |
| Mazzucca 2017 [58] | To test a theoretically driven | ECEC | Int: 92 children; 13 services | 10 weeks | PA: Minutes in MVPA per hour; | No significant differences were noted in the amount of MVPA (p |

| | | | | | | |
|---|---|---|---|---|---|---|
| USA<br>Cluster RCT | intervention using professional development to facilitate changes to the physical and social environment to improve PA outcomes for children in ECE services | SES NR | <br>Con: 90 children; 13 services | Provide opportunities for children to be physically active; offer educator training to provide safe and developmentally appropriate PA; educators to promote the benefits of PA with children; limit the time children spend sitting; create a physical environment that promotes PA<br><br>Face to face; telephone; online; written; SMS<br><br>Research team; childcare staff<br><br>Usual care | Accelerometer (ActiGraph GT3X); Wear time: 5 days, valid if 4 hours/day for 3 days; Cut points: Evenson et al 2008[44]; Pate et al 2006[30]<br><br>10 weeks | = 0.33) between intervention and control at follow-up |
| Mehtälä 2017 [7]<br>Finland<br>Cluster RCT | To evaluate the effect of the 12-month cluster randomized HIPPA intervention on the everyday PA of children between the ages of 4 to 5 years | ECEC + home<br><br>SES NR | Int: 56 children; 7 services<br><br>Con: 46 children; 7 services | 12 months<br><br>Provide opportunities for children to be physically active; offer educator training to provide safe and developmentally appropriate PA; limit use of screen time; create a physical environment that promotes PA; parental involvement<br><br>Face to face; written<br><br>Research team; childcare staff | PA: Minutes in MVPA per weekday; Accelerometer (Actigraph GT3X); Wear time: 5 days, valid if 8 hours/day for 3 days including 1 weekend day; Cut points: van Cauwenberghe et al 2011[59]<br><br>13-15 months | Children in the intervention engaged in significantly more MVPA at follow-up than the control group did ($p = 0.016$) |

| | | | | | | |
|---|---|---|---|---|---|---|
| | | | | Usual care | | |
| O'Dwyer 2013 [60]<br>UK (England)<br>Cluster RCT | To investigate the effect of a school-based active play intervention on preschool children's sedentary time and PA | ECEC<br><br>Low SES and/or marginalised population | Int: 109 children; 6 services<br><br>Con: 131 children; 6 services | 6 weeks<br><br>Provide opportunities for children to be physically active; offer educator training to provide safe and developmentally appropriate PA; educators to promote the benefits of PA with children<br><br>Face to face<br><br>Childcare staff; other org/expert<br><br>Usual care | PA: MVPA (mins per day); Accelerometer (ActiGraph GT1M); Wear time: 7 days, valid if 3 days including 1 weekend day; Cut points: Sirard et al 2005[27]<br><br>6 weeks | No significant intervention effects were observed for MVPA (mins per day) at follow-up (p value NR) |
| Okely 2020 [19]<br>Australia<br>Cluster RCT | To test the 6-month effects of an 18-month intervention on PA in ECEC settings in low income communities | ECEC + home<br><br>Low SES and/or marginalised population | Int: 348 children; 22 services<br><br>Con: 310 children; 21 services | 18 months<br><br>Provide opportunities for children to be physically active; offer educator training to provide safe and developmentally appropriate PA; educators to promote the benefits of PA with children; limit the time children spend sitting; parental involvement<br><br>Face to face; telephone; online; written | PA: Mins/hour spent in MVPA during childcare; Accelerometer (Actigraph GT1M, GT3X+ and GT3X); Wear time: 1 week, valid if 3 hours for 1 day (childcare hours); Cut points: Pate et al 2006[30]<br><br>Adverse events: number of adverse events; NR<br><br>6 months | There were no significant differences between groups in change of mins/hour spent in MVPA ($p = 0.861$)<br><br>No adverse events were reported |

| | | | | | | |
|---|---|---|---|---|---|---|
| | | | | Childcare staff; other org/expert<br><br>Usual care | | |
| Palmer 2019 [61]<br>USA<br>Cluster RCT | To examine changes in motor skills and PA elicited by a 5-week, 600-min motor skill intervention | ECEC<br><br>Low SES and/or marginalised population | Int: 64 children; 6 services<br><br>Con: 38 children; 4 services | 5 weeks<br><br>Provide opportunities for children to be physically active<br><br>Face to face<br><br>Research team<br><br>Usual care | PA: Minutes of MVPA during outdoor free play; Accelerometer (Actigraph GT3X); Wear time: 7 days; Cut points: Evenson et al 2008[44]<br><br>FMS: total motor skills; TGMD-3 total score<br><br>5 weeks | No significant group differences at follow-up for MVPA (p = 0.08)<br><br>Children in the intervention from baseline scored significantly higher on all motor skills assessments at follow-up compared with the control group (p < 0.001) |
| Pate 2016 [2]<br>USA<br>Cluster RCT | To test the effects of an adaptable ecologic preschool intervention, implemented by preschool teachers, on the PA of young children | ECEC<br><br>SES NR | Int: 249 children; 8 services<br><br>Con: 239 children; 8 services | 31 weeks<br><br>Provide opportunities for children to be physically active; offer educator training to provide safe and developmentally appropriate PA; educators to promote the benefits of PA with children; create a physical environment that promotes PA<br><br>Face to face; written<br><br>Research team; childcare staff<br><br>Waitlist control | PA: Mins/hour of MVPA during the preschool day; Accelerometer (ActiGraph GT1M and GT3X); Wear time: 5 days, valid if 50% of school hours for 3 days; Cut points: Pate et al 2006[30]<br><br>31 weeks | Children in the intervention engaged in significantly more MVPA than children in control at follow-up (p = 0.01) |

| | | | | | | |
|---|---|---|---|---|---|---|
| Peden 2022 [6]<br>Australia<br>Stepped wedge cluster RCT | To evaluate the efficacy of a 'blended' professional learning program for early childhood educators on the PA levels of children in ECEC services. | ECEC + home<br><br>SES NR | Group 1: 87 children; 5 services<br><br>Group 2: 112 children; 5 services<br><br>Group 3: 105 children; 3 services | 12 weeks<br><br>Provide opportunities for children to be physically active; develop and adopt policies for PA and PA education programs; offer educator training to provide safe and developmentally appropriate PA; educators to promote the benefits of PA with children; limit the time children spend sitting; create a physical environment that promotes PA; parental involvement<br><br>Face to face; online; written<br><br>Research team; childcare staff<br><br>Usual care | PA: % of time spent in MVPA during care; Accelerometer (ActiGraph GT1M and GT3X+); Wear time: Valid if 3 hours/1 day; Cut points: Pate et al 2006[30]<br><br>Adverse events: number of adverse events; NR<br><br>12 weeks | There were no significant differences between groups in % of time spent in MVPA (p = 0.80)<br><br>There were no adverse events |
| Puder 2011 [62]<br>Switzerland<br>Cluster RCT | To test the effect of a multidimensional lifestyle intervention on aerobic fitness and adiposity in predominantly migrant preschool children | ECEC + home<br><br>Low SES and/or marginalised population | Int: 342 children; 20 classrooms<br><br>Con: 310 children; 20 classrooms<br><br>30 schools | 1 school year<br><br>Provide opportunities for children to be physically active; develop and adopt policies for PA and PA education programs; offer educator training to provide safe and developmentally appropriate PA; educators to promote the benefits of PA | PA: Total PA (counts/min); Accelerometer (ActiGraph MTI/CSA 7164); Wear time: 5 days, valid if 6 hours/day for 2 weekdays and 1 weekend day; Cut points: Not reported | No significant group differences in total PA (p = 0.54), BMI (p = 0.31), QoL (p = 0.17), dynamic balance (p = 0.35) or spatial working memory (p = 0.58)<br><br>No adverse events occurred |

| | | | | | | |
|---|---|---|---|---|---|---|
| | | | | with children; limit the use of screen time; support healthy sleeping habits; create a physical environment that promotes PA; parental involvement<br><br>Face to face; written; CD<br><br>Childcare staff; other org/expert<br><br>Usual care | Weight/anthropometrics: BMI; Electronic scale<br><br>Wellbeing: HRQoL; PedsQL 4.0<br><br>FMS: (dynamic balance); Balance beam<br><br>Cognitive functioning: Spatial working memory; Intelligence and development scales (IDS)<br><br>Adverse events: adverse events during intervention sessions; NR<br><br>40 weeks | |
| Ray 2020 [63]<br>Finland<br>Cluster RCT | To examine the effects of a preschool-based family-involving multicomponent intervention on children's energy balance-related behaviours (EBRBs) | ECEC + home<br><br>SES NR | Int: 361 children; 13 services<br><br>Con: 441 children; 19 services | 23 weeks<br><br>Provide opportunities for children to be physically active; offer educator training to provide safe and developmentally appropriate PA; educators to promote the benefits of PA with children; limit the use of screen time; parental involvement | PA: Total PA (min/day); Accelerometer (ActiGraph wGT3X-BT); Wear time: 7 days, valid if 10 hours/day for 4 days (including 1 weekend day); Cut points: Evenson et al 2008[44]<br><br>Cognitive functioning: cognitive self-regulation | At follow-up, no differences were detected in total PA (min/day) (p = 0.858) or cognitive (p = 0.505) and emotional (p = 0.405) self-regulation skills between the intervention and control group |

| | | | | | | |
|---|---|---|---|---|---|---|
| | | | | Face to face; online; written<br><br>Research team; childcare staff<br><br>Waitlist control | skills; Cognitive SR skills sub-dimension of the Child social behaviour questionnaire<br><br>Wellbeing: emotional self-regulation skills; Emotional SR skills sub-dimension of the child social behaviour questionnaire<br><br>24-28 weeks | |
| Razak 2018 [3] Australia Cluster RCT | To assess the efficacy of scheduling multiple periods of outdoor free-play in increasing the time children spend in MVPA while attending childcare | ECEC<br><br>Low + high SES | Int: 171 children; 5 services<br><br>Con: 268 children; 5 services | 3 months<br><br>Provide opportunities for children to be physically active<br><br>Face to face; telephone; written<br><br>Research team; childcare staff; other org/expert<br><br>Usual care | PA: MVPA (mins) while in care per day; Accelerometer (Actigraph GT3X+); Wear time: 5 days, valid if 50% wear time/1day; Cut points: Pate et al 2006[30]<br><br>Adverse events: Number of child injuries; Survey<br><br>12 weeks | Mean daily minutes of MVPA in care significantly increased at follow-up among children attending intervention services ($p = 0.03$) compared to control<br><br>No significant difference in the number of injuries reported across the study period between groups ($p = 1.0$) |
| Reilly 2006 [13] UK (Scotland) Cluster RCT | To assess whether a PA intervention reduces body mass index in young children | ECEC + home<br><br>SES NR | Int: 268 children; 18 services<br><br>Con: 277 children; 18 services | 24 weeks<br><br>Provide opportunities for children to be physically active; offer educator training to provide safe and | PA: % time in MVPA; Accelerometer (ActiGraph MTI/CSA 7164); Wear time: 6 days; Cut points: Puyau et al 2004[40] | Group was marginally significant for % time in MVPA (mean value being greater in control nurseries, $p = 0.05$), and was not a significant effect in the model for BMI SD score ($p = 0.87$) |

| | | | | | | |
|---|---|---|---|---|---|---|
| | | | | developmentally appropriate PA; limit the use of screen time; create a physical environment that promotes PA; parental involvement<br><br>Face to face; written<br><br>Research team; childcare staff<br><br>Usual care | Weight/anthropometrics: BMI SD score; Portable stadiometer & scales<br><br>FMS: Global motor skills score; Movement assessment battery<br><br>24 weeks | The intervention group had significantly higher performance in movement skills tests than the control group (p = 0.0027) |
| Roth 2015 [20] Germany Cluster RCT | To evaluate a multi-component preschool intervention program led by preschool teachers to enhance PA and motor skill performance in 4- and 5-year old children | ECEC + home<br><br>Low + high SES | Int: 368 children; 21 services<br><br>Con: 341 children; 20 services | 11 months<br><br>Provide opportunities for children to be physically active; offer educator training to provide safe and developmentally appropriate PA; educators to promote the benefits of PA with children; limit the use of screen time; parental involvement<br><br>Face to face; written<br><br>Research team; childcare staff; other org/expert<br><br>Usual care | PA: % time spent in MVPA; Accelerometer (ActiGraph GT1M); Wear time: 7 days, valid if 7 hours/day for 3 weekdays and 1 weekend day; Cut points: Pate et al 2006[30]<br><br>FMS: motor skill proficiency; Motor skills composite score<br><br>Weight/anthropometrics: BMI (centile); Height and weight<br><br>Adverse events: accidents; Parental questionnaire<br><br>52-60 weeks | No significant effect of intervention on MVPA (p = 0.859) or BMI (p = 0.949) at follow-up<br><br>Significant increase in children's motor skills performance in favour of the intervention (p = 0.007)<br><br>No change in rate of accidents (p = 0.273) |

| | | | | | | |
|---|---|---|---|---|---|---|
| Sharp 2017 [64]<br>UK (Wales)<br>Cluster RCT | To evaluate the effectiveness of a multi-component intervention to increase pre-school children's PA levels and consumption levels of target fruit and vegetables | ECEC + home<br><br>SES NR | Int: 88 children; 2 services<br><br>Con: 84 children; 2 services | 3 months<br><br>Provide opportunities for children to be physically active; educators to promote the benefits of PA with children; parental involvement<br><br>Face to face; online; written; video<br><br>Research team; childcare staff<br><br>Usual care | PA: Total in-school step count; Accelerometer (FitBit Zip); Wear time: 10 days (2 school weeks); Cut points: N/A<br><br>Weight/anthropometrics: BMI score; Stadiometer and calibrated digital scales<br><br>2 months | No significant group differences in total in-school step count (p = 0.10) or BMI score were found between baseline and follow-up (p value NR) |
| Szpunar 2021 [5]<br>Canada<br>Cluster RCT | To examine the effectiveness of an evidence-based, stakeholder-informed, written PA and sedentary time policy on young children's movement behaviours | ECEC<br><br>SES NR | Int: 100 children; 5 services<br><br>Con: 122 children; 4 services | 8 weeks<br><br>Develop and adopt policies for PA and PA education programs; offer educator training to provide safe and developmentally appropriate PA<br><br>Face to face; written<br><br>Research team; childcare staff<br><br>Waitlist control | PA: Mins/hour spent in MVPA; Accelerometer (ActiGraph GT3x-BT); Wear time: 5 days, valid if 5 hours/day for 2 days; Cut points: Pate et al 2006[30]<br><br>9 weeks | No significant effect of intervention on change in MVPA from baseline (p = 0.52) |
| Telford 2021 [65]<br>Australia<br>Cluster RCT | To evaluate the effect of a multisite and pragmatic peer coach-based | ECEC<br><br>Low + high SES | Int: 170 children; 8 services | 6 months<br><br>Provide opportunities for children to be physically | PA: Mins/hour spent in MVPA; Accelerometer (ActiGraph GT3x); Wear time: 3 days, valid if 3 | Significant intervention effect for change in MVPA (p < .001) from baseline, and no change in BMI score (p value NR) |

| | | | | | | |
|---|---|---|---|---|---|---|
| | intervention on the level of PA of the children attending childcare services | | Con: 144 children; 7 services | active; offer educator training to provide safe and developmentally appropriate PA; educators to promote the benefits of PA with children<br><br>Face to face; written<br><br>Research team; childcare staff; other org/expert<br><br>Usual care | hours/day; Cut points: Pate et al 2006[30]<br><br>Weight/anthropometrics: BMI score; portable stadiometer and digital scales<br><br>6 months | |
| Trost 2008 [11]<br>USA<br>Cluster RCT | To evaluate the effect of a 'move and learn' curriculum on PA in 3-to 5-year-olds attending a half-day preschool program | ECEC<br><br>SES NR | Int: 2 classrooms<br><br>Con: 2 classrooms<br><br>1 service, 42 children (Int vs Con NR) | 8 weeks<br><br>Provide opportunities for children to be physically active; offer educator training to provide safe and developmentally appropriate PA; educators to promote the benefits of PA with children; create a physical environment that promotes PA<br><br>Face to face; video<br><br>Childcare staff; other org/expert<br><br>Usual care | PA: MVPA minutes; Accelerometer (ActiGraph WAM 7164); Wear time: 2 days/week for 10 weeks; Cut points: Sirard et al 2005[27]<br><br>8 weeks | MVPA was significantly greater for the intervention group compared to control at follow-up ($p < 0.05$) |
| Tucker 2017 [18]<br>Canada | To examine the effectiveness of the SPACE intervention | ECEC<br><br>SES NR | Int: 200 children; 11 services | 8 weeks | PA: Mins/hour spent in MVPA; Accelerometer (Actical Z and B series); | MVPA significantly increased among children in the |

| | | | | | | |
|---|---|---|---|---|---|---|
| Cluster RCT | on preschoolers' PA levels and sedentary time during childcare hours | | Con: 138 children; 11 services | Provide opportunities for children to be physically active; offer educator training to provide safe and developmentally appropriate PA; create a physical environment that promotes PA<br><br>Face to face<br><br>Research team; childcare staff; other org/expert<br><br>Usual care | Wear time: 5 days, valid if 5hours/day for 2 days; Cut points: Adolph et al 2012[24]<br><br>8 weeks | intervention compared to control (p = 0.002) |
| Vaughn 2021 [66]<br>USA<br>Cluster RCT | To evaluate the effectiveness of a ECE-based nutrition and PA social marketing intervention | ECEC + home<br><br>SES NR | Int: 200 children; 11 services<br><br>Con: 138 children; 11 services | 8 months<br><br>Provide opportunities for children to be physically active; offer educator training to provide safe and developmentally appropriate PA; educators to promote the benefits of PA with children; parental involvement<br><br>Face to face; online; written<br><br>Research team; childcare staff<br><br>Waitlist control | PA: Non-sedentary PA min/day; Accelerometer (ActiGraph GT3X+); Wear time: 7 days, valid if 6 hours/day for 3 days; Cut points: Evenson et al 2008[44]; Pate et al 2006[30]; Troiano et al 2008[67]<br><br>Weight/anthropometrics: BMI z-score; Stadiometer and digital scale<br><br>8-10 months | No significant difference in change from baseline to follow-up in non-sedentary PA (p = 0.439) or BMI z-score (p = 0.679) between intervention and control |
| Wadsworth 2020 [68] | To determine which type of | ECEC | Int 1 FMS: 25 children; 2 classrooms | 7 weeks | PA: % of time spent in MVPA (male/female); | Males in the control group spent significantly less time in MVPA |

| | | | | | | |
|---|---|---|---|---|---|---|
| USA<br>Cluster RCT | intervention, PA or FMS focus, elicits MVPA during outdoor play | Low SES and/or marginalised population | Int 2 PA: 25 children; 2 classrooms<br><br>Int 3 FMS+PA: 25 children; 2 classrooms<br><br>Con: 23 children; 2 classrooms<br><br>1 service | Int 1,2,3: Provide opportunities for children to be physically active; educators to promote the benefits of PA with children; create a physical environment that promotes PA<br><br>Face to face<br><br>Childcare staff<br><br>Usual care | Accelerometer (Actigraph GT3X); Wear time: 2 days/week for 7 weeks; Cut points: Butte et al 2014[33]<br><br>9 weeks | compared to the males in the FMS group ($p < 0.05$), the FMS + PA group ($p < 0.01$), and the PA group ($p < 0.05$)<br><br>For females, the difference in MVPA between the control and the intervention groups were not significant ($p > 0.05$) |
| Wolfenden 2019 [69]<br>Australia<br>Cluster RCT | To assess the efficacy of a childcare based intervention in increasing child PA by allowing children access to outdoor areas for free-play when a structured activity is not taking place. | ECEC<br><br>Low + high SES | Int: 165 children; 3 services<br><br>Con: 185 children; 3 services | 3 months<br><br>Provide opportunities for children to be physically active; develop and adopt policies for PA and PA education programs<br><br>Face to face<br><br>Childcare staff; other org/expert<br><br>Usual care | PA: % of time spent in MVPA during care; Accelerometer (Actigraph GT3X+); Wear time: 1-5 days (days in care); Cut points: Pate et al 2006[30]<br><br>Cognitive functioning: Inhibition; Early Years Toolbox ('Go/No-Go' game)<br><br>3 months | No significant differences between groups in changes in % of time spent in MVPA ($p = 0.14$), nor on measures of child cognition (inhibition $p = 0.45$) |

SES: socioeconomic status; PA: physical activity; MVPA: moderate to vigorous PA; TPA: total PA; TGMD-2/3: Test of Gross Movement Development, 2$^{nd}$/3$^{rd}$ edition; LMS: locomotor skills; NR: not reported; HRQoL: health related quality of life; GMS: gross motor skills; GMQ: gross motor quotient; FMS: fundamental movement skills; CD: compact disc; DVD: digital versatile disc

Table S4: PRISMA checklist

| Section and Topic | Item # | Checklist item | Location where item is reported |
|---|---|---|---|
| **TITLE** | | | |
| Title | 1 | Identify the report as a systematic review. | Title page |
| **ABSTRACT** | | | |
| Abstract | 2 | See the PRISMA 2020 for Abstracts checklist. | Page 2<br>See checklist below |
| **INTRODUCTION** | | | |
| Rationale | 3 | Describe the rationale for the review in the context of existing knowledge. | Page 3-5 |
| Objectives | 4 | Provide an explicit statement of the objective(s) or question(s) the review addresses. | Page 5 |
| **METHODS** | | | |
| Eligibility criteria | 5 | Specify the inclusion and exclusion criteria for the review and how studies were grouped for the syntheses. | Page 6-9 |
| Information sources | 6 | Specify all databases, registers, websites, organisations, reference lists and other sources searched or consulted to identify studies. Specify the date when each source was last searched or consulted. | Page 9<br>Supporting information S2 |
| Search strategy | 7 | Present the full search strategies for all databases, registers and websites, including any filters and limits used. | Supporting information S2 |
| Selection process | 8 | Specify the methods used to decide whether a study met the inclusion criteria of the review, including how many reviewers screened each record and each report retrieved, whether they worked independently, and if applicable, details of automation tools used in the process. | Page 10 |
| Data collection process | 9 | Specify the methods used to collect data from reports, including how many reviewers collected data from each report, whether they worked independently, any processes for obtaining or confirming data from study investigators, and if applicable, details of automation tools used in the process. | Page 10-11 |
| Data items | 10a | List and define all outcomes for which data were sought. Specify whether all results that were compatible with each outcome domain in each study were sought (e.g. for all measures, time points, analyses), and if not, the methods used to decide which results to collect. | Page 10-11 |
| | 10b | List and define all other variables for which data were sought (e.g. participant and intervention characteristics, funding sources). Describe any assumptions made about any missing or unclear information. | Page 10-11 |
| Study risk of bias assessment | 11 | Specify the methods used to assess risk of bias in the included studies, including details of the tool(s) used, how many reviewers assessed each study and whether they worked independently, and if applicable, details of automation tools used in the process. | Page 11-12 |

| | | | |
|---|---|---|---|
| Effect measures | 12 | Specify for each outcome the effect measure(s) (e.g. risk ratio, mean difference) used in the synthesis or presentation of results. | Page 12-13 |
| Synthesis methods | 13a | Describe the processes used to decide which studies were eligible for each synthesis (e.g. tabulating the study intervention characteristics and comparing against the planned groups for each synthesis (item #5)). | Page 12, 13 |
| | 13b | Describe any methods required to prepare the data for presentation or synthesis, such as handling of missing summary statistics, or data conversions. | Page 12-14 |
| | 13c | Describe any methods used to tabulate or visually display results of individual studies and syntheses. | Page 15 |
| | 13d | Describe any methods used to synthesize results and provide a rationale for the choice(s). If meta-analysis was performed, describe the model(s), method(s) to identify the presence and extent of statistical heterogeneity, and software package(s) used. | Page 12, 15 |
| | 13e | Describe any methods used to explore possible causes of heterogeneity among study results (e.g. subgroup analysis, meta-regression). | Page 14-15 |
| | 13f | Describe any sensitivity analyses conducted to assess robustness of the synthesized results. | Page 15-16 |
| Reporting bias assessment | 14 | Describe any methods used to assess risk of bias due to missing results in a synthesis (arising from reporting biases). | Page 15-16 |
| Certainty assessment | 15 | Describe any methods used to assess certainty (or confidence) in the body of evidence for an outcome. | N/R |
| **RESULTS** | | | |
| Study selection | 16a | Describe the results of the search and selection process, from the number of records identified in the search to the number of studies included in the review, ideally using a flow diagram. | Page 15 |
| | 16b | Cite studies that might appear to meet the inclusion criteria, but which were excluded, and explain why they were excluded. | Page 15-16 |
| Study characteristics | 17 | Cite each included study and present its characteristics. | Page 16-20<br>Table S3 |
| Risk of bias in studies | 18 | Present assessments of risk of bias for each included study. | Page 20<br>Figure 2 |
| Results of individual studies | 19 | For all outcomes, present, for each study: (a) summary statistics for each group (where appropriate) and (b) an effect estimate and its precision (e.g. confidence/credible interval), ideally using structured tables or plots. | Page 21-27<br>Figures 3-7 |
| Results of syntheses | 20a | For each synthesis, briefly summarise the characteristics and risk of bias among contributing studies. | Page 21-28 |
| | 20b | Present results of all statistical syntheses conducted. If meta-analysis was done, present for each the summary estimate and its precision (e.g. confidence/credible interval) and measures of statistical heterogeneity. If comparing groups, describe the direction of the effect. | Page 21-27 |
| | 20c | Present results of all investigations of possible causes of heterogeneity among study results. | Page 21-27 |
| | 20d | Present results of all sensitivity analyses conducted to assess the robustness of the synthesized results. | Page 21-27 |
| Reporting biases | 21 | Present assessments of risk of bias due to missing results (arising from reporting biases) for each synthesis assessed. | N/R |
| Certainty of evidence | 22 | Present assessments of certainty (or confidence) in the body of evidence for each outcome assessed. | N/R |
| **DISCUSSION** | | | |

| | | | |
|---|---|---|---|
| Discussion | 23a | Provide a general interpretation of the results in the context of other evidence. | Page 27-29 |
| | 23b | Discuss any limitations of the evidence included in the review. | Page 29-30 |
| | 23c | Discuss any limitations of the review processes used. | Page 29-30 |
| | 23d | Discuss implications of the results for practice, policy, and future research. | Page 30-31 |
| **OTHER INFORMATION** | | | |
| Registration and protocol | 24a | Provide registration information for the review, including register name and registration number, or state that the review was not registered. | Page 32 |
| | 24b | Indicate where the review protocol can be accessed, or state that a protocol was not prepared. | Page 32 |
| | 24c | Describe and explain any amendments to information provided at registration or in the protocol. | N/A |
| Support | 25 | Describe sources of financial or non-financial support for the review, and the role of the funders or sponsors in the review. | Page 32 |
| Competing interests | 26 | Declare any competing interests of review authors. | Page 32 |
| Availability of data, code and other materials | 27 | Report which of the following are publicly available and where they can be found: template data collection forms; data extracted from included studies; data used for all analyses; analytic code; any other materials used in the review. | Page 32 |

## PRISMA for abstracts checklist

| Section and Topic | Item # | Checklist item | Reported (Yes/No) |
|---|---|---|---|
| **TITLE** | | | |
| Title | 1 | Identify the report as a systematic review. | Yes |
| **BACKGROUND** | | | |
| Objectives | 2 | Provide an explicit statement of the main objective(s) or question(s) the review addresses. | Yes |
| **METHODS** | | | |
| Eligibility criteria | 3 | Specify the inclusion and exclusion criteria for the review. | Inclusion |
| Information sources | 4 | Specify the information sources (e.g. databases, registers) used to identify studies and the date when each was last searched. | Yes |
| Risk of bias | 5 | Specify the methods used to assess risk of bias in the included studies. | Yes |
| Synthesis of results | 6 | Specify the methods used to present and synthesise results. | Yes |
| **RESULTS** | | | |
| Included studies | 7 | Give the total number of included studies and participants and summarise relevant characteristics of studies. | Yes (not participants) |

| | | | |
|---|---|---|---|
| Synthesis of results | 8 | Present results for main outcomes, preferably indicating the number of included studies and participants for each. If meta-analysis was done, report the summary estimate and confidence/credible interval. If comparing groups, indicate the direction of the effect (i.e. which group is favoured). | Yes |
| **DISCUSSION** | | | |
| Limitations of evidence | 9 | Provide a brief summary of the limitations of the evidence included in the review (e.g. study risk of bias, inconsistency and imprecision). | No (word limit) |
| Interpretation | 10 | Provide a general interpretation of the results and important implications. | Yes |
| **OTHER** | | | |
| Funding | 11 | Specify the primary source of funding for the review. | In main manuscript |
| Registration | 12 | Provide the register name and registration number. | No<br>osf-registrations-vy3nb-v1 |

**References**


1. Jackson JK, Jones J, Nguyen H, et al. Obesity prevention within the early childhood education and care setting: A systematic review of dietary behavior and physical activity policies and guidelines in high income countries. *Int J Environ Res Public Health*. 2021; 18(2). doi:10.3390/ijerph18020838
2. Pate RR, Brown WH, Pfeiffer KA, et al. An intervention to increase physical activity in children: A randomized controlled trial with 4-year-olds in preschools. *Am J Prev Med*. 2016; 51(1): 12-22. doi:https://dx.doi.org/10.1016/j.amepre.2015.12.003
3. Razak LA, Yoong SL, Wiggers J, et al. Impact of scheduling multiple outdoor free-play periods in childcare on child moderate-to-vigorous physical activity: A cluster randomised trial. *Int J Behav Nutr Phys Act*. 2018; 15(1): 34. doi:10.1186/s12966-018-0665-5
4. Engel A, Broderick C, van Doorn N, et al. Effect of a fundamental motor skills intervention on fundamental motor skill and physical activity in a preschool setting: A cluster randomized controlled trial. *Pediatr Exerc Sci*. 2022; 34(2): 57-66. doi:10.1123/pes.2021-0021
5. Szpunar M, Driediger M, Johnson AM, et al. Impact of the Childcare Physical Activity (PLAY) policy on young children's physical activity and sedentary time: A pilot clustered randomized controlled trial. *Int J Environ Res Public Health*. 2021; 18(14): 13. doi:https://dx.doi.org/10.3390/ijerph18147468
6. Peden M, Eady M, Okely A, Patterson K, Batterham M, Jones R. A blended professional learning intervention for early childhood educators to target the promotion of physical activity and healthy eating: The HOPPEL cluster randomized stepped-wedge trial. *BMC Public Health*. 2022; 22(1): 1353. doi:10.1186/s12889-022-13542-w
7. Mehtälä MA, Sääkslahti A, Soini A, et al. The effect of the cluster randomized HIPPA intervention on childcare children's overall physical activity. *Balt J Health Phys Act*. 2017; 9(4): 89-111. doi: 10.29359/BJHPA.09.4.08
8. Goldfield GS, Harvey ALJ, Grattan KP, et al. Effects of child care intervention on physical activity and body composition. *Am J Prev Med*. 2016; 51(2): 225-231. doi:https://dx.doi.org/10.1016/j.amepre.2016.03.024
9. Finch M, Wolfenden L, Morgan PJ, Freund M, Jones J, Wiggers J. A cluster randomized trial of a multi-level intervention, delivered by service staff, to increase physical activity of children attending center-based childcare. *Prev Med*. 2014; 58: 9-16. doi:10.1016/j.ypmed.2013.10.004

10. Chow AF, Leis A, Humbert L, Muhajarine N, Engler-Stringer R. Healthy Start-Départ Santé: A pilot study of a multilevel intervention to increase physical activity, fundamental movement skills and healthy eating in rural childcare centres. *Can J Public Health*. 2016; 107(3): e312-e318. doi:10.17269/cjph.107.5279
11. Trost SG, Fees B, Dzewaltowski D. Feasibility and efficacy of a "move and learn" physical activity curriculum in preschool children. *J Phys Act Health*. 2008; 5(1): 88-103. doi:10.1123/jpah.5.1.88
12. Malden S, Reilly JJ, Gibson AM, et al. A feasibility cluster randomised controlled trial of a preschool obesity prevention intervention: ToyBox-Scotland. *Pilot Feasibility Stud*. 2019; 5: 128. doi:10.1186/s40814-019-0521-7
13. Reilly JJ, Kelly L, Montgomery C, et al. Physical activity to prevent obesity in young children: Cluster randomised controlled trial. *BMJ*. 18 2006;333(7577):1041. doi:10.1136/bmj.38979.623773.55
14. Alhassan S, St Laurent CW, Burkart S, Greever CJ, Ahmadi MN. Feasibility of integrating physical activity into early education learning standards on preschooler's physical activity levels. *J Phys Act Health*. 2019; 16(2): 101-107. doi:https://dx.doi.org/10.1123/jpah.2017-0628
15. Kipping R, Langford R, Brockman R, et al. Child-care self-assessment to improve physical activity, oral health and nutrition for 2-to 4-year-olds: A feasibility cluster RCT. *Public Health Res*. 2019; 7(13): 1-164. doi:10.3310/phr07130
16. Bonvin A, Barral J, Kakebeeke TH, et al. Effect of a governmentally-led physical activity program on motor skills in young children attending child care centers: A cluster randomized controlled trial. *Int J Behav Nutr Phys Act*. 2013; 10: 90. doi:10.1186/1479-5868-10-90
17. Alhassan S, Sirard JR, Robinson TN. The effects of increasing outdoor play time on physical activity in Latino preschool children. *Int J Pediatr Obes*. 2007; 2(3): 153-8. doi:10.1080/17477160701520108
18. Tucker P, Vanderloo LM, Johnson AM, et al. Impact of the Supporting Physical Activity in the Childcare Environment (SPACE) intervention on preschoolers' physical activity levels and sedentary time: A single-blind cluster randomized controlled trial. *Int J Behav Nutr Phys Act*. 2017; 14(1): 120. doi:10.1186/s12966-017-0579-7
19. Okely AD, Stanley RM, Jones RA, et al. 'Jump start' childcare-based intervention to promote physical activity in pre-schoolers: Six-month findings from a cluster randomised trial. *Int J Behav Nutr Phys Act*. 2020; 17(1): 6. doi:10.1186/s12966-020-0910-6

20. Roth K, Kriemler S, Lehmacher W, Ruf KC, Graf C, Hebestreit H. Effects of a physical activity intervention in preschool children. *Med Sci Sports Exerc*. 2015; 47(12): 2542-51. doi:https://dx.doi.org/10.1249/MSS.0000000000000703
21. Adamo KB, Wasenius NS, Grattan KP, et al. Effects of a preschool intervention on physical activity and body composition. *J Pediatr*. 2017; 188: 42-49.e2. doi:https://dx.doi.org/10.1016/j.jpeds.2017.05.082
22. Nagpal TS, Goldfield GS, da Silva DF, et al. The effects of intervening with physical activity in the early years (ages 3-5) on health-related quality of life: A secondary analysis of the Activity Begins in Childhood (ABC) trial. *Qual Life Res*. 2021; 30(1): 221-227. doi:https://dx.doi.org/10.1007/s11136-020-02587-2
23. Wasenius NS, Grattan KP, Harvey ALJ, Naylor PJ, Goldfield GS, Adamo KB. The effect of a physical activity intervention on preschoolers' fundamental motor skills - A cluster RCT. *J Sci Med Sport*. 2018; 21(7): 714-719. doi:10.1016/j.jsams.2017.11.004
24. Adolph AL, Puyau MR, Vohra FA, Nicklas TA, Zakeri IF, Butte NF. Validation of uniaxial and triaxial accelerometers for the assessment of physical activity in preschool children. *J Phys Act Health*. 2012; 9(7): 944-53. doi:10.1123/jpah.9.7.944
25. Aivazidis D, Venetsanou F, Aggeloussis N, Gourgoulis V, Kambas A. Enhancing motor competence and physical activity in kindergarten. *J Phys Act Health*. 2019; 16(3): 184-190. doi:https://dx.doi.org/10.1123/jpah.2018-0260
26. De Craemer M, De Decker E, De Bourdeaudhuij I, Verloigne M, Manios Y, Cardon G. The translation of preschoolers' physical activity guidelines into a daily step count target. *J Sports Sci*. 2015; 33(10): 1051-1057. doi:10.1080/02640414.2014.981850
27. Sirard JR, Trost SG, Pfeiffer KA, Dowda M, Pate RR. Calibration and evaluation of an objective measure of physical activity in preschool children. *J Phys Act Health*. 2005; 2(3): 345-357. doi:10.1123/jpah.2.3.345
28. Alhassan S, Nwaokelemeh O, Ghazarian M, Roberts J, Mendoza A, Shitole S. Effects of locomotor skill program on minority preschoolers' physical activity levels. *Pediatr Exerc Sci*. 2012; 24(3): 435-49. doi:10.1123/pes.24.3.435
29. Alhassan S, Nwaokelemeh O, Lyden K, Goldsby T, Mendoza A. A pilot study to examine the effect of additional structured outdoor playtime on preschoolers' physical activity levels. *Child Care Pract*. 2013; 19(1): 23-35. doi:10.1080/13575279.2012.712034
30. Pate RR, Almeida MJ, McIver KL, Pfeiffer KA, Dowda M. Validation and calibration of an accelerometer in preschool children. *Obesity (Silver Spring)*. 2006; 14(11): 2000-6. doi:10.1038/oby.2006.234

31. Alhassan S, Sudarsky L, Dangol G, et al. Feasibility and preliminary efficacy of a childcare provider-led activity intervention on toddlers' physical activity levels: A pilot randomized controlled study. *Child Care Pract*. 2022; 1-15. doi:https://dx.doi.org/10.1080/13575279.2022.2082381
32. Andersen E, Ovreas S, Jorgensen KA, Borch-Jenssen J, Moser T. Children's physical activity level and sedentary behaviour in Norwegian early childhood education and care: effects of a staff-led cluster-randomised controlled trial. *BMC Public Health*. 2020;20(1):1651. doi:https://dx.doi.org/10.1186/s12889-020-09725-y
33. Butte NF, Wong WW, Lee JS, Adolph AL, Puyau MR, Zakeri IF. Prediction of energy expenditure and physical activity in preschoolers. *Med Sci Sports Exerc*. Jun 2014;46(6):1216-26. doi:10.1249/mss.0000000000000209
34. Annesi JJ, Smith AE, Tennant GA. Effects of the Start For Life treatment on physical activity in primarily African American preschool children of ages 3-5 years. *Psychol Health Med*. 2013; 18(3): 300-9. doi:10.1080/13548506.2012.712704
35. Pate RR, Pfeiffer KA, Trost SG, Ziegler P, Dowda M. Physical activity among children attending preschools. *Pediatrics*. 2004; 114(5): 1258-63. doi:10.1542/peds.2003-1088-L
36. Bellows LL, Davies PL, Anderson J, Kennedy C. Effectiveness of a physical activity intervention for Head Start preschoolers: A randomized intervention study. *Am J Occup Ther*. 2013; 67(1): 28-36. doi:10.5014/ajot.2013.005777
37. Byun W, Lau EY, Brusseau TA. Feasibility and effectiveness of a wearable technology-based physical activity intervention in preschoolers: A pilot study. *Int J Environ Res Public Health*. 2018; 15(9): 23. doi:https://dx.doi.org/10.3390/ijerph15091821
38. Cardon G, Labarque V, Smits D, De Bourdeaudhuij I. Promoting physical activity at the pre-school playground: The effects of providing markings and play equipment. *Prev Med*. 2009; 48(4): 335-40. doi:10.1016/j.ypmed.2009.02.013
39. Carroll AV, Spring KE, Wadsworth DD. The effect of a teacher-guided and -led indoor preschool physical activity intervention: A feasibility study. *Early Child Educ J*. 2022; 50, 1475–1483. doi: 10.1007/s10643-021-01274-2
40. Puyau MR, Adolph AL, Vohra FA, Zakeri I, Butte NF. Prediction of activity energy expenditure using accelerometers in children. *Med Sci Sports Exerc*. 2004; 36(9): 1625-31. doi: 10.1249/01.MSS.0000139898.30804.60

41. De Bock F, Genser B, Raat H, Fischer JE, Renz-Polster H. A participatory physical activity intervention in preschools: A cluster randomized controlled trial. *Am J Prev Med*. 2013; 45(1): 64-74. doi:10.1016/j.amepre.2013.01.032
42. De Bock F, Menze J, Becker S, Litaker D, Fischer J, Seidel I. Combining accelerometry and HR for assessing preschoolers' physical activity. *Med Sci Sports Exerc*. 2010; 42(12): 2237-43. doi:10.1249/MSS.0b013e3181e27b5d
43. De Craemer M, De Decker E, Verloigne M, De Bourdeaudhuij I, Manios Y, Cardon G. The effect of a kindergarten-based, family-involved intervention on objectively measured physical activity in Belgian preschool boys and girls of high and low SES: The ToyBox-study. *Int J Behav Nutr Phys Act*. 2014; 11(1): 38. doi:10.1186/1479-5868-11-38
44. Evenson KR, Catellier DJ, Gill K, Ondrak KS, McMurray RG. Calibration of two objective measures of physical activity for children. *J Sports Sci*. 2008; 26(14): 1557-65. doi:10.1080/02640410802334196
45. De Craemer M, Verloigne M, De Bourdeaudhuij I, et al. Effect and process evaluation of a kindergarten-based, family-involved cluster randomised controlled trial in six European countries on four- to six-year-old children's steps per day: The ToyBox-study. *Int J Behav Nutr Phys Act*. 2017; 14(1): 116. doi:10.1186/s12966-017-0574-z
46. Driediger M, Truelove S, Johnson AM, et al. The impact of shorter, more frequent outdoor play periods on preschoolers' physical activity during childcare: A cluster randomized controlled trial. *Int J Environ Res Public Health*. 2019; 16(21): 26. doi:https://dx.doi.org/10.3390/ijerph16214126
47. Duff C, Issartel J, O'Brien W, Belton S. Kids active: Evaluation of an educator-led active play and fundamental movement skill intervention in the Irish preschool setting. *J Mot Learn Dev*. 2019; 7(3): 389-407. doi:https://dx.doi.org/10.1123/jmld.2018-0039
48. España-Romero V, Mitchell JA, Dowda M, O'Neill JR, Pate RR. Objectively measured sedentary time, physical activity and markers of body fat in preschool children. *Pediatr Exerc Sci*. 2013; 25(1): 154-163. doi:10.1123/pes.25.1.154
49. Eliakim A, Nemet D, Balakirski Y, Epstein Y. The effects of nutritional-physical activity school-based intervention on fatness and fitness in preschool children. *J Pediatr Endocrinol Metab*. 2007; 20(6): 711-8. doi:10.1515/jpem.2007.20.6.711
50. Ellis YG, Cliff DP, Howard SJ, Okely AD. Feasibility, acceptability, and potential efficacy of a childcare-based intervention to reduce sitting time among pre-schoolers: A pilot randomised controlled trial. *J Sports Sci*. 2019; 37(2): 146-155. doi:https://dx.doi.org/10.1080/02640414.2018.1486362

51. Fitzgibbon ML, Stolley MR, Schiffer LA, et al. Hip-Hop to Health Jr. Obesity Prevention Effectiveness Trial: Postintervention results. *Obesity (Silver Spring)*. 2011; 19(5): 994-1003. doi: 10.1038/oby.2010.314

52. Pfeiffer KA, McIver KL, Dowda M, Almeida MJCA, Pate RR. Validation and calibration of the actical accelerometer in preschool children. *Med Sci Sports Exerc*. 2006; 38(1): 152-157. doi:10.1249/01.mss.0000183219.44127.e7

53. Hoffman JA, Schmidt EM, Arguello DJ, et al. Online preschool teacher training to promote physical activity in young children: A pilot cluster randomized controlled trial. *Sch Psychol*. 2020; 35(2): 118-127. doi:10.1037/spq0000349

54. Jones RA, Riethmuller A, Hesketh K, Trezise J, Batterham M, Okely AD. Promoting fundamental movement skill development and physical activity in early childhood settings: A cluster randomized controlled trial. *Pediatr Exerc Sci*. 2011; 23(4): 600-15. doi:10.1123/pes.23.4.600

55. Jones RA, Okely AD, Hinkley T, Batterham M, Burke C. Promoting gross motor skills and physical activity in childcare: A translational randomized controlled trial. *J Sci Med Sport*. 2016; 19(9): 744-9. doi:https://dx.doi.org/10.1016/j.jsams.2015.10.006

56. Leis A, Ward S, Vatanparast H, et al. Effectiveness of the Healthy Start-Départ Santé approach on physical activity, healthy eating and fundamental movement skills of preschoolers attending childcare centres: A randomized controlled trial. *BMC Public Health*. 2020; 20(1): 1-12. doi: 10.1186/s12889-020-08621-9

57. Mavilidi MF, Rigoutsos S, Venetsanou F. Training early childhood educators to promote children's physical activity. *Early Child Educ J*. 2022; 50, 785–794. doi:10.1007/s10643-021-01191-4

58. Mazzucca SL. *Physical Activity and Sedentary Behavior in Early Care and Education Centers: Identifying Opportunities and Testing Strategies to Support Active Classroom Environments*. [PhD thesis]. Chapel Hill, NC: The University of North Carolina at Chapel Hill; 2017. doi.org/10.17615/wjcx-ww07

59. van Cauwenberghe E, Labarque V, Trost SG, de Bourdeaudhuij I, Cardon G. Calibration and comparison of accelerometer cut points in preschool children. *Int J Pediatr Obes*. 2011; 6(2-2): e582-9. doi:10.3109/17477166.2010.526223

60. O'Dwyer MV, Fairclough SJ, Ridgers ND, Knowles ZR, Foweather L, Stratton G. Effect of a school-based active play intervention on sedentary time and physical activity in preschool children. *Health Educ Res*. 2013; 28(6): 931-42. doi:10.1093/her/cyt097

61. Palmer KK, Chinn KM, Robinson LE. The effect of the CHAMP intervention on fundamental motor skills and outdoor physical activity in preschoolers. *J Sport Health Sci*. 2019; 8(2): 98-105. doi:10.1016/j.jshs.2018.12.003

62. Puder JJ, Marques-Vidal P, Schindler C, et al. Effect of multidimensional lifestyle intervention on fitness and adiposity in predominantly migrant preschool children (Ballabeina): Cluster randomised controlled trial. *BMJ*. 2011; 343: d6195. doi:10.1136/bmj.d6195

63. Ray C, Figuereido R, Vepsäläinen H, et al. Effects of the preschool-based family-involving DAGIS intervention program on children’s energy balance-related behaviors and self-regulation skills: A clustered randomized controlled trial. *Nutrients*. 2020; 12(9): 2599. doi: 10.3390/nu12092599

64. Sharp CA. *Development and Evaluation of a Healthy Eating and Physical Activity Behaviour Change Intervention Targeting 3-4 year old Children at School, Extending to the Home*. [PhD Thesis]. Gwynedd, North Wales, UK: Bangor University; 2017. Accessed August 13, 2024 https://research.bangor.ac.uk/portal/en/theses/development-and-evaluation-of-a-healthy-eating-and-physical-activity-behaviour-change-intervention-targeting-3--4-year-old-children-at-school-extending-to-the-home(13127e97-1312-4149-86d6-96bf732e07d8).html

65. Telford RM, Olive LS, Telford RD. A peer coach intervention in childcare centres enhances early childhood physical activity: The Active Early Learning (AEL) cluster randomised controlled trial. *Int J Behav Nutr Phys Act*. 2021; 18(1): 37. doi:10.1186/s12966-021-01101-2

66. Vaughn AE, Hennink-Kaminski H, Moore R, et al. Evaluating a child care-based social marketing approach for improving children's diet and physical activity: Results from the Healthy Me, Healthy We cluster-randomized controlled trial. *Transl Behav Med*. 2021; 11(3): 775-784. doi:10.1093/tbm/ibaa113

67. Troiano RP, Berrigan D, Dodd KW, Mâsse LC, Tilert T, McDowell M. Physical activity in the United States measured by accelerometer. *Med Sci Sports Exerc*. 2008; 40(1): 181-8. doi:10.1249/mss.0b013e31815a51b3

68. Wadsworth DD, Johnson JL, Carroll AV, Pangelinan MM, Rudisill ME, Sassi J. Intervention strategies to elicit MVPA in preschoolers during outdoor play. *Int J Environ Res Public Health*. 2020; 17(2): 19. doi:https://dx.doi.org/10.3390/ijerph17020650

69. Wolfenden L, Jones J, Parmenter B, et al. Efficacy of a free-play intervention to increase physical activity during childcare: A randomized controlled trial. *Health Educ Res*. 2019; 34(1): 84-97. doi:https://dx.doi.org/10.1093/her/cyy041
